# Leishmania donovani Impedes Antileishmanial Immunity by Suppressing Dendritic Cells via the TIM-3 Receptor

**DOI:** 10.1128/mbio.03309-21

**Published:** 2022-08-04

**Authors:** Md. Naushad Akhtar, Sahil Kumar, Pradip Sen

**Affiliations:** a Division of Cell Biology and Immunology, Council of Scientific and Industrial Research-Institute of Microbial Technology, Chandigarh, India; National Institute of Allergy and Infectious Diseases

**Keywords:** dendritic cells, TIM-3, visceral leishmaniasis, *Leishmania donovani*, immunoregulation, NF-κB

## Abstract

An immunological hallmark of visceral leishmaniasis (VL), caused by Leishmania donovani, is profound immunosuppression. However, the molecular basis for this immune dysfunction has remained ill defined. Since dendritic cells (DCs) normally initiate antileishmanial immune responses, we investigated whether DCs are dysregulated during L. donovani infection and assessed its role in immunosuppression. Accordingly, we determined the regulatory effect of L. donovani on DCs. Notably, it is still unclear whether L. donovani activates or suppresses DCs. In addition, the molecular mechanism and the relevant receptor (or receptors) mediating the immunoregulatory effect of L. donovani on DCs are largely undefined. Here, we report that L. donovani inhibited DC activation/maturation by transmitting inhibitory signals through the T cell immunoglobulin and mucin protein-3 (TIM-3) receptor and thereby suppressed antileishmanial immune responses. L. donovani in fact triggered TIM-3 phosphorylation in DCs, which in turn recruited and activated a nonreceptor tyrosine kinase, Btk. Btk then inhibited DC activation/maturation by suppressing the NF-κB pathway in an interleukin-10 (IL-10)-dependent manner. Treatment with TIM-3-specific blocking antibody or suppressed expression of TIM-3 or downstream effector Btk made DCs resistant to the inhibitory effects of L. donovani. Adoptive transfer experiments further demonstrated that TIM-3-mediated L. donovani-induced inhibition of DCs plays a crucial role in the suppression of the antileishmanial immune response *in vivo*. These findings identify TIM-3 as a new regulator of the antileishmanial immune response and demonstrate a unique mechanism for host immunosuppression associated with L. donovani infection.

## INTRODUCTION

Visceral leishmaniasis (VL), caused by Leishmania donovani (LD), is potentially a fatal disease that annually affects 0.2 to 0.4 million people worldwide (1, 2). One of the immunopathological consequences of VL (especially in the later stages) is marked immunosuppression that increases the chance of secondary infections and consequent fatal outcomes (3–5). The immunosuppression stems from T cell dysfunction and/or increased suppressive activity of regulatory T (Treg) cells, which in turn are largely controlled by dendritic cells (DCs) (6–10). In fact, during leishmaniasis, DCs play a crucial role in initiating and regulating antileishmanial T cell reactivity (10–12). Accordingly, the inhibition of DCs upon virulent *Leishmania* infection is thought to impede the priming of CD4^+^ T cells and subsequent host-protective T-helper 1 (Th1) immunity (13). Although various *Leishmania* species have been shown to inhibit DCs (14–19), it is not yet clear whether L. donovani promotes or inhibits DC activation and maturation. In addition, the key receptor and the molecular events mediating the immunoregulatory effect of L. donovani on DCs are ill defined. Such information is critically required to understand the molecular basis for immunosuppression and consequent disease pathogenesis during L. donovani infection.

Over the past decade, the receptor T cell immunoglobulin and mucin protein-3 (TIM-3) has emerged as a critical regulator of immune function (20). TIM-3 was initially identified as a Th1-specific surface protein (21). Later on, TIM-3 was found to be expressed by DCs (22). In DCs, TIM-3 has been shown by us to inhibit the activation and maturation processes by blocking the nuclear factor κB (NF-κB) signaling pathway (23). Until now, only a few studies have demonstrated the role for DC-derived TIM-3 in immune regulation and that too primarily focused in the context of antitumor immunity. For example, we and others have shown that c-Src-mediated enhancement of TIM-3 expression on tumor-associated DCs attenuates antitumor immunity (24, 25). In addition, TIM-3 has been shown to regulate the antitumor function of intratumoral CD103^+^ DCs (26).

Despite being a key immune receptor, the involvement of TIM-3 in leishmaniasis is still understudied. So far, a few reports have only depicted an increased TIM-3 expression on CD8^+^ T cells in VL and cutaneous leishmaniasis (CL) patients (27, 28). In contrast, another group has demonstrated an increased *HAVCR2* mRNA (which encodes TIM-3) expression in the liver and a reduced *HAVCR2* mRNA expression in the spleen of L. donovani-infected mice (29). However, the role of TIM-3 in immunobiology of leishmaniasis has remained undefined. Since TIM-3 is also expressed by DCs (22), we set out to determine the role of TIM-3 in L. donovani-mediated regulation of DC activity. Specifically, in this study we investigated whether L. donovani influences the activation and maturation of DCs; if so, then whether TIM-3 has any role in this process; and how TIM-3 mediates the regulatory effect of L. donovani on DCs. In addition, we finally examined whether TIM-3, by regulating DC activity, influences the host immune response against L. donovani.

## RESULTS

### L. donovani infection inhibits DC activation and maturation *in vitro* by suppressing NF-κB signaling.

**FIG 1:**
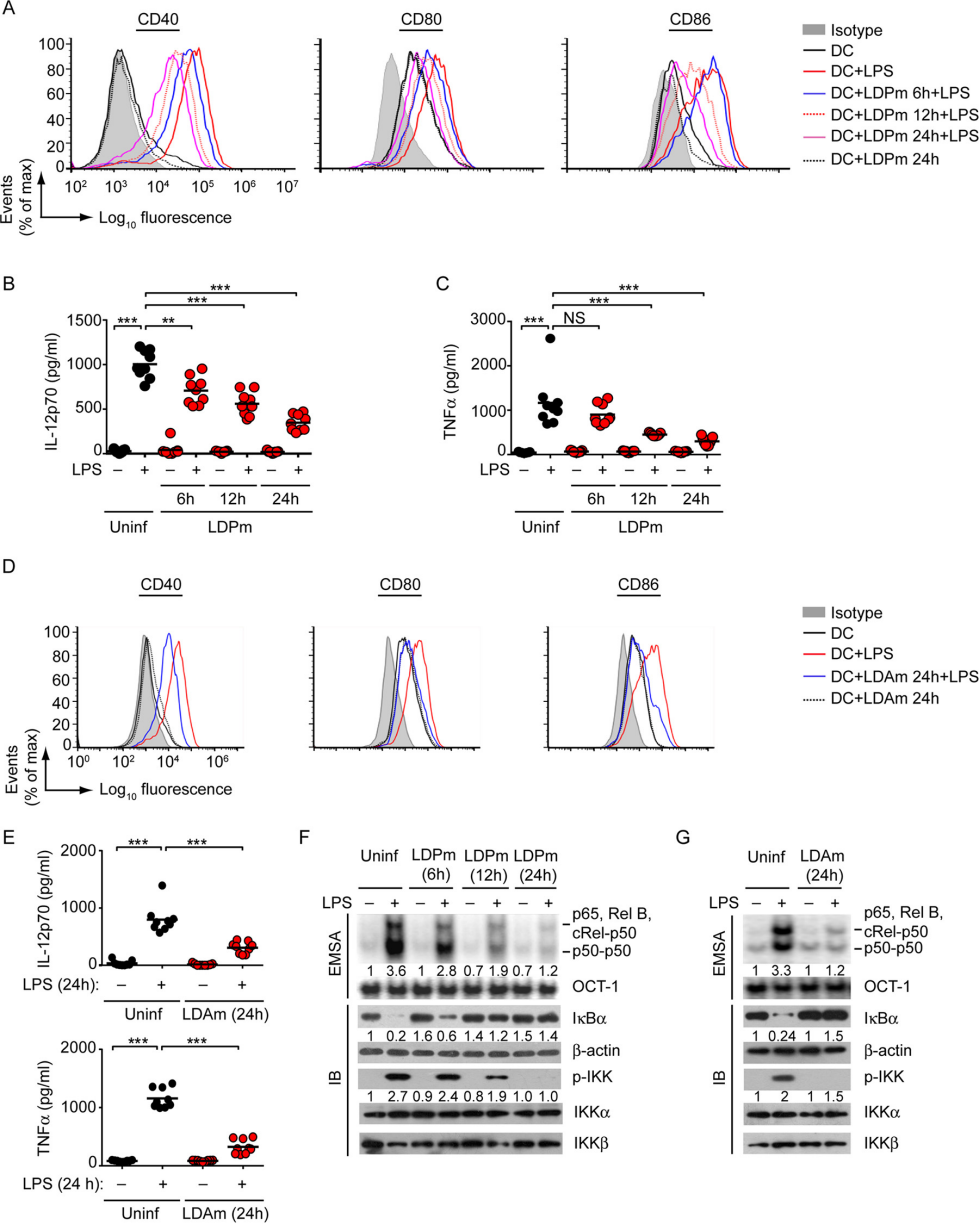
L. donovani suppresses the NF-κB pathway and BMDC activation and maturation. (A) BMDCs were infected with L. donovani promastigotes (LDPm) for the specified times or left uninfected (DC) and then treated with LPS for 24 h. Expression of costimulatory molecules on BMDCs was evaluated by flow cytometry. Isotype, isotype-matched control antibody. (B and C) ELISA measuring IL-12p70 (B) and TNF-α (C) secretion from BMDCs infected with LDPm for 6 to 24 h or left uninfected (Uninf) and then cultured with (+) or without (−) LPS for 24 h. (D) Flow cytometry analysis of costimulatory molecule expression on BMDCs kept uninfected (DCs) or infected with L. donovani amastigotes (LDAm) for 24 h and then cultured (for 24 h) with LPS. (E) Secretion of IL-12p70 (upper panel) and TNF-α (lower panel) by BMDCs that had been infected with LDAm and treated with LPS as in panel D was assessed via ELISA. “Uninf” represents uninfected BMDCs. (F and G) BMDCs were left uninfected or infected with LDPm (F) or LDAm (G) for indicated times and then treated with or without LPS for 0.5 h. The DNA-binding activity of nuclear NF-κB or OCT-1 (internal control) was assessed by EMSA, and the expression of IκBα, phosphorylated (p-) IKK, and the loading controls such as β-actin, IKKα, and IKKβ was measured by immunoblot (IB) analysis. The subunits of bound NF-κB complexes were earlier confirmed by supershift analysis (70). Numbers below lanes indicate densitometry of NF-κB binding (normalized to OCT-1 binding) and the level of IκBα (normalized to β-actin) and phosphorylated IKK (normalized to mean of IKKα and IKKβ levels), presented relative to that of uninfected BMDCs given no LPS treatment. Data in panels A, D, F, and G are representative of three independent analyses. Compiled data on relative mean fluorescence intensities of costimulatory molecule expression for panels A and D and densitometry results for panels F and G from three separate experiments are shown in Fig. S1. Data shown in panels B, C, and E are a compilation of three separate experiments (*n *= 3 per experiment). Each symbol depicts data of individual replicates, and bars indicate means. ***, *P* < 0.001; **, *P* < 0.01; NS, not significant.

As mentioned above, there is a debate as to whether L. donovani infection promotes or inhibits DC activation and maturation (14–19). Accordingly, we first clarified this aspect. We infected bone marrow-derived dendritic cells (BMDCs), generated from BALB/c mice, with L. donovani promastigotes (LDPm; the extracellular form of L. donovani) for various times (6 h, 12 h, and 24 h) and stimulated them with lipopolysaccharide (LPS) for 24 h. We then determined the activation and maturation of BMDCs by analyzing the expression of costimulatory molecules CD40, CD80, and CD86 via flow cytometry and the secretion of proinflammatory cytokines interleukin-12 (IL-12) and tumor necrosis factor alpha (TNF-α) via enzyme-linked immunosorbent assay (ELISA). After treatment with LPS, the expression of CD40, CD80, and CD86 was substantially upregulated on BMDCs (Fig. 1A; also see Fig. S1A in the supplemental material). However, infection of BMDCs with LDPm led to a gradual decrease in LPS-stimulated CD40, CD80, and CD86 expression, with peak inhibition observed at 24 h postinfection (Fig. 1A; Fig. S1A). Corroborating costimulatory molecule expression data, LPS treatment greatly enhanced IL-12 and TNF-α secretion from BMDCs (Fig. 1B and C). However, this LPS-induced IL-12 and TNF-α secretion was progressively inhibited with increasing time of LDPm infection (maximum inhibition occurred at 24 h postinfection; Fig. 1B and C). Our results depicting these inhibitory effects of LDPm on DCs align with previous studies (14, 15, 30, 31). In addition, consistent with an earlier report (30), we observed that LDPm alone (without LPS treatment) could not trigger any proinflammatory cytokine secretion or upregulate costimulatory molecule expression by BMDCs (Fig. 1A to C; Fig. S1A). Next, we tested whether L. donovani amastigotes (LDAm; the intracellular form of L. donovani) exhibit a similar inhibitory effect on DCs. To verify this issue, we infected BMDCs with LDAm for 24 h before stimulation with LPS, because at 24 h of LDPm infection we found maximum DC inhibition (Fig. 1A; Fig. S1A). Here again, we found that LDAm largely attenuated LPS-stimulated upregulation of costimulatory molecule expression and proinflammatory cytokine secretion by BMDCs (Fig. 1D and E; Fig. S1B). Collectively, these results suggest that L. donovani suppresses the activation and maturation of BMDCs.

10.1128/mbio.03309-21.1 FIG S1 Supporting information for Fig. 1. (A) Related to Fig. 1A. BMDCs were infected (or not) with LDPm for the specified times and treated with LPS for 24 h, and costimulatory molecule (CD40, CD80, and CD86) expression was assessed via flow cytometry (Fig. 1A). The mean fluorescence intensities (MFIs) of costimulatory molecule expression were measured after subtracting nonspecific background signal (isotype control) and are expressed here as fold change relative to uninfected DCs given no LPS treatment. The combined data of three separate analyses are presented here. (B) Related to Fig. 1D. Graphs depict the data pooled from three separate experiments for the expression of costimulatory molecules on BMDCs infected with LDAm for 24 h or left uninfected and then cultured with or without LPS for 24 h. Representative flow cytometry data have been shown in Fig. 1D. The MFI values were measured as in panel A and presented here as fold change relative to uninfected BMDCs cultured without LPS. (C and D) Related to Fig. 1F and G. Graphs show combined densitometry data of three separate experiments for NF-κB DNA-binding activity, and the level of IκBα and phosphorylated IKK (shown in Fig. 1F and G) in BMDCs infected with LDPm (C) or LDAm (D) for indicated times or kept uninfected and subsequently cultured (for 0.5 h) with or without LPS. Densitometry quantification was performed as described in Fig. 1F and presented relative to uninfected BMDCs cultured without LPS. In all panels, error bars indicate standard deviations (SD), and each symbol in the graphs corresponds to data derived from one independent experiment. ***, *P* < 0.001; **, *P* < 0.01; *, *P* < 0.05; NS, not significant. Download FIG S1, TIF file, 0.5 MB. Copyright © 2022 Akhtar et al. 2022 Akhtar et al. https://creativecommons.org/licenses/by/4.0/ This content is distributed under the terms of the Creative Commons Attribution 4.0 International license.

We then sought to determine the molecular basis for L. donovani-induced DC suppression. Previous reports have demonstrated that the transcription factor NF-κB serves a major role in regulating the activation and maturation of DCs (32, 33). In fact, inhibition of NF-κB alone can lead to DC suppression (33–35). Accordingly, we determined the ability of L. donovani to regulate NF-κB signaling in DCs. We infected BMDCs with LDPm for various times, stimulated them with LPS, and then analyzed the DNA-binding activity of NF-κB and the degradation of inhibitory IκBα protein by electrophoretic mobility shift assay (EMSA) and immunoblot analysis, respectively. Additionally, we determined the activation of upstream IκB kinase (IKK) by measuring its phosphorylation via immunoblot analysis. Within 12 h postinfection, LDPm started inhibiting LPS-stimulated NF-κB DNA binding, IκBα degradation, and IKK phosphorylation, and by 24 h these LPS-induced events were almost blocked (Fig. 1F; Fig. S1C). Likewise, LDAm inhibited LPS-induced NF-κB signaling in BMDCs (Fig. 1G; Fig. S1D). We have recently demonstrated that L. donovani infection does not alter the level of toll-like receptor 4 (TLR4; an LPS receptor) expression on DCs (10). The latter report ruled out the possibility that L. donovani suppressed NF-κB activation by downregulating TLR4 expression and thereby promoting hyporesponsiveness of DCs to LPS stimulation. Together, these results suggest that L. donovani impedes NF-κB signaling, leading to the suppression of BMDCs.

### TIM-3 is necessary for L. donovani-induced inhibition of DC activation and maturation *in vitro*.

**FIG 2:**
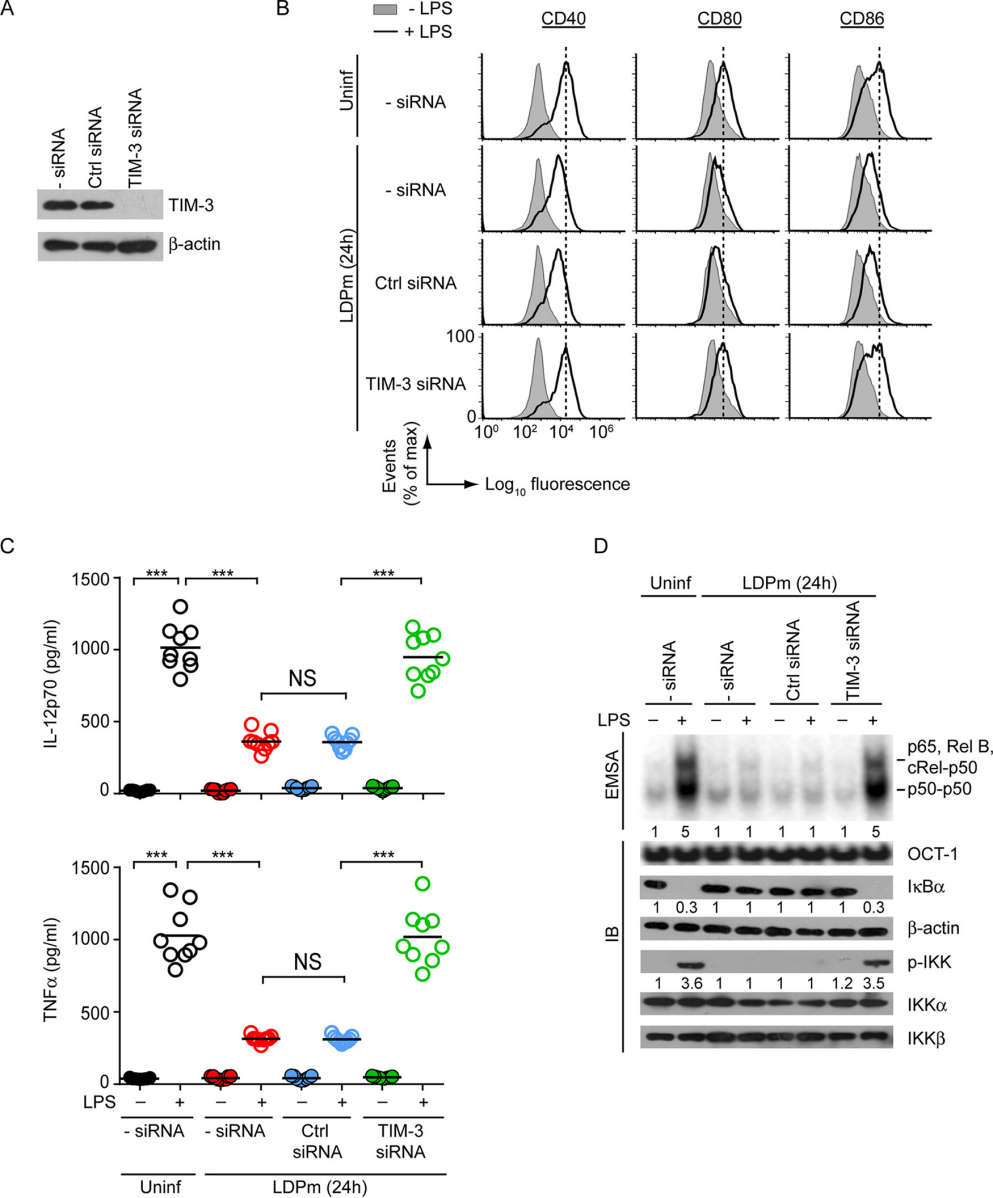
TIM-3 is necessary for L. donovani-induced inhibition of BMDCs. (A) Expression of TIM-3 and β-actin (loading control) in BMDCs transfected with no siRNA (− siRNA), control siRNA (Ctrl siRNA), or TIM-3-specific siRNA, determined by immunoblotting (representative blot of three separate experiments). (B and C) Analysis of the effect of TIM-3 silencing on costimulatory molecule expression (by flow cytometry) (B) and IL-12p70 and TNF-α secretion (via ELISA) (C) by BMDCs that were infected with LDPm for 24 h or left uninfected and subsequently stimulated with LPS for 24 h. Data are representative of three independent experiments (B) (see Fig. S2A for compiled mean fluorescence intensity data from three experiments) or pooled from three individual experiments (C) (*n *= 3 per experiment). Each symbol in panel C shows data of individual replicate, and bars indicate means. (D) BMDCs were transfected as in panel A and then infected for 24 h with LDPm or left uninfected and cultured for 0.5 h with or without LPS. Nuclear NF-κB or OCT-1 DNA binding was analyzed by EMSA, and the expression of indicated proteins was measured by immunoblotting (representative data of three independent experiments). Numbers below lanes, densitometry (as in Fig. 1F), presented relative to control BMDCs (i.e., BMDCs left untransfected [− siRNA], uninfected, and cultured without LPS). Compiled densitometry results of three experiments are presented in Fig. S2B. ***, *P* < 0.001; NS, not significant.

Next, we directed our effort to identify the potential receptor that contributed to L. donovani-mediated inhibition of DCs. One such receptor appeared to be TIM-3 because TIM-3 is expressed by DCs and plays a key role in the downregulation of DC activation and maturation (22, 23). Furthermore, the expression of TIM-3 has been shown to be increased in VL patients, albeit on CD8^+^ T cells (27). These reports tempted us to verify the involvement of TIM-3, if any, in L. donovani-induced BMDC inhibition. Accordingly, we silenced TIM-3 expression using small interfering RNAs (siRNAs) (Fig. 2A) and analyzed its effect on L. donovani-mediated regulation of BMDC activation and maturation. We observed that LDPm effectively suppressed LPS-induced upregulation of costimulatory molecule expression and proinflammatory cytokine secretion by control BMDCs (i.e., untransfected or control siRNA-transfected BMDCs) (Fig. 2B and C; Fig. S2A). These suppressive effects of LDPm on BMDCs, however, were blocked by TIM-3 silencing (Fig. 2B and C; Fig. S2A). These experiments depicted an indispensable role for TIM-3 in L. donovani-mediated suppression of BMDCs.

10.1128/mbio.03309-21.2 FIG S2 Supporting information for Fig. 2. (A) Related to Fig. 2B. Bar graphs show the compiled data of three separate experiments for MFI of costimulatory molecule expression on BMDCs that were treated with specified siRNAs, infected for 24 h with LDPm or left uninfected, and cultured with or without LPS for 24 h. MFIs were determined as mentioned in Fig. S1A and presented as fold change compared to control BMDCs (BMDCs kept untransfected and uninfected and cultured without LPS). The corresponding flow cytometry data have been shown in Fig. 2B. (B) Related to Fig. 2D. Bar graphs show combined densitometry data of three separate experiments evaluating the intensity of NF-κB DNA binding and the levels of IκBα and phosphorylated IKK (Fig. 2D) in BMDCs transfected with indicated siRNA, then infected with LDPm for 24 h or kept uninfected, and cultured (for 0.5 h) with or without LPS. The relative densitometry quantification was performed as mentioned in Fig. 1F. In all panels, error bars indicate SD and each symbol in the graphs corresponds to data derived from one independent experiment. ***, *P* < 0.001; **, *P* < 0.01; *, *P* < 0.05; NS, not significant. Download FIG S2, TIF file, 0.7 MB. Copyright © 2022 Akhtar et al. 2022 Akhtar et al. https://creativecommons.org/licenses/by/4.0/ This content is distributed under the terms of the Creative Commons Attribution 4.0 International license.

To verify whether TIM-3 promoted L. donovani-induced inhibition of BMDCs by preventing NF-κB signaling, we monitored the effect of TIM-3 silencing on NF-κB activation in L. donovani-infected BMDCs. We found that silencing of TIM-3 effectively blocked the inhibitory effect of LDPm on LPS-induced NF-κB DNA-binding activity, IκBα degradation, and IKK phosphorylation in BMDCs (Fig. 2D; Fig. S2B). Together, these *in vitro* results demonstrate that TIM-3 is essential for mediating the inhibitory effects of L. donovani on NF-κB signaling and subsequent activation and maturation of BMDCs.

### L. donovani infection-associated impairment of sDC function.

**FIG 3:**
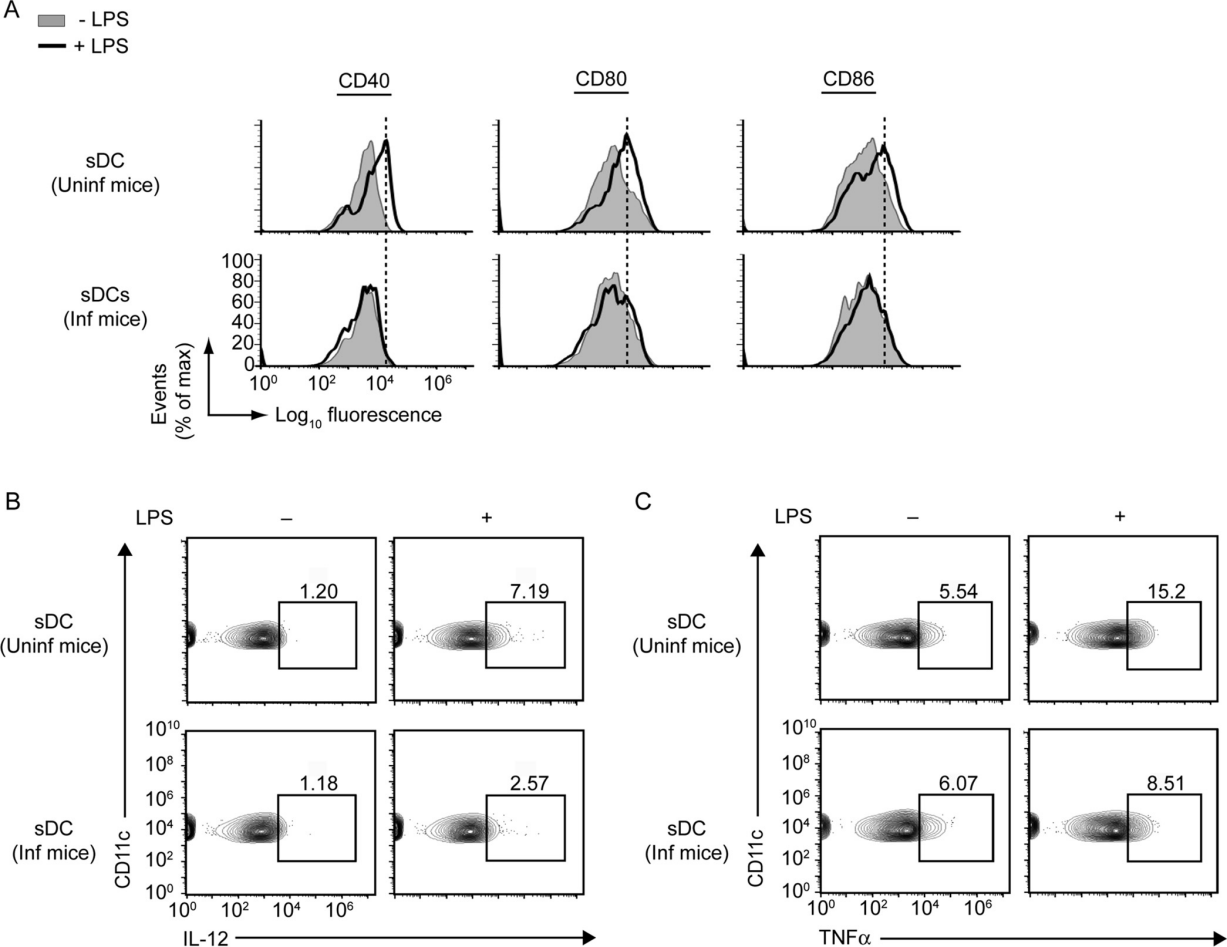
sDCs are inhibited during L. donovani infection. (A) Spleens were isolated from uninfected (Uninf) mice or mice infected (Inf) with L. donovani for 45 days. Splenocytes were then prepared and cultured in the presence (+) or absence (−) of LPS for 24 h. Expression of costimulatory molecules on sDCs (i.e., CD11c^+^ F4/80^−^ gated cells; see Fig. S3A for gating strategy) was assessed via flow cytometry. Results are representative of three analyses. The relative mean fluorescence intensities of costimulatory molecule expression from three different analyses are presented in Fig. S3B. (B and C) Splenocytes of uninfected and 45-day-infected mice were cultured with LPS as in panel A. The frequency of sDCs producing IL-12 (B) and TNF-α (C) was determined via flow cytometry. Numbers in outlined areas represent the percentage of sDCs expressing IL-12 or TNF-α. Gating strategies for flow cytometry analysis are illustrated in Fig. S3C and E. Results are representative of three independent analyses. Compiled data for three separate experiments are presented in Fig. S3D and F.

As we found with BMDCs that L. donovani inhibited the activation and maturation of BMDCs and that TIM-3 was necessary to promote such inhibition (Fig. 1 and 2; Fig. S1 and S2), we next verified whether L. donovani exhibits a similar inhibitory effect on splenic DCs (sDCs), and if so, whether TIM-3 serves any role in mediating this process. Accordingly, we first determined whether L. donovani infection in mice influences the activation and maturation of sDCs *in vivo*. For this purpose, we isolated splenocytes from uninfected and day 45-infected mice and treated these splenocytes with LPS for 24 h. We then analyzed the expression of costimulatory molecules and proinflammatory cytokines by sDCs (defined as CD11c^+^ F4/80^−^ cells throughout this study [8]) via flow cytometry. We found that sDCs from L. donovani-infected mice, compared to those from uninfected mice, showed lower expression of costimulatory molecules and proinflammatory cytokines despite LPS stimulation (Fig. 3; Fig. S3). These results point to an impairment of sDC activation and maturation consequent to L. donovani infection.

10.1128/mbio.03309-21.3 FIG S3 Supporting information for Fig. 3. (A) Gating strategy related to Fig. 3A. Splenocytes from uninfected and 45-day-infected mice were cultured with or without LPS for 24 h. The costimulatory molecule expression by sDCs (assessed via flow cytometry) has been presented in Fig. 3A. The corresponding gating strategy is presented here. Briefly, splenocytes were gated based on forward scatter (FSC), side scatter (SSC), and lack of F4/80 expression. Subsequently, F4/80-negative cells expressing CD11c (i.e., CD11c^+^ F4/80^−^ cells; defined as sDCs) were examined for CD40, CD80, and CD86 expression via flow cytometry (shown in Fig. 3A). (B) Related to Fig. 3A. Splenocytes of uninfected (Uninf) and 45-day-infected (Inf) mice were cultured in the presence or absence of LPS for 24 h. Expression of costimulatory molecules on sDCs [i.e., CD11c^+^ F4/80^−^ gated cells; gating strategy has been described in panel A) was analyzed by flow cytometry and has been presented in Fig. 3A. The respective MFI values for costimulatory molecule expression (calculated as in Fig. S1A) are presented here (bar graphs) as fold change relative to sDCs that were derived from uninfected mice and subsequently cultured without LPS. Results shown here depict combined data of three individual analyses. (C) Gating strategy related to Fig. 3B. Splenocytes were gated as in panel A. Then, gated CD11c^+^ F4/80^−^ cells were examined for IL-12^+^ population (shown in Fig. 3B) based on background staining with isotype control antibody. (D) Related to Fig. 3B. Graph shows compiled data (of three experiments) of the percentage of IL-12-expressing sDCs (from uninfected or 45-day-infected mice) following LPS stimulation for 24 h (details given in Fig. 3B legend). (E) Gating strategy related to Fig. 3C. Splenocytes were gated as in panel A. The CD11c^+^ F4/80^−^ gated cells (i.e., sDCs) were then examined (via flow cytometry) for TNF-α^+^ population (shown in Fig. 3C) based on background staining with isotype control antibody. (F) Related to Fig. 3C. Graph shows compiled data (of three independent experiments) of the percentage of TNF-α-expressing sDCs that were detected after culturing splenocytes of uninfected and 45-day-infected mice in the presence (+) and absence (−) of LPS for 24 h. Corresponding flow cytometry data have been shown in Fig. 3C. In all panels, error bars indicate SD, and each symbol in the graphs corresponds to data derived from one independent experiment. ***, *P* < 0.001; **, *P* < 0.01; *, *P* < 0.05. Download FIG S3, TIF file, 0.8 MB. Copyright © 2022 Akhtar et al. 2022 Akhtar et al. https://creativecommons.org/licenses/by/4.0/ This content is distributed under the terms of the Creative Commons Attribution 4.0 International license.

### *In vivo* TIM-3 blockade restores the activation/maturation capacity of sDCs in L. donovani-infected mice and promotes parasite clearance.

**FIG 4:**
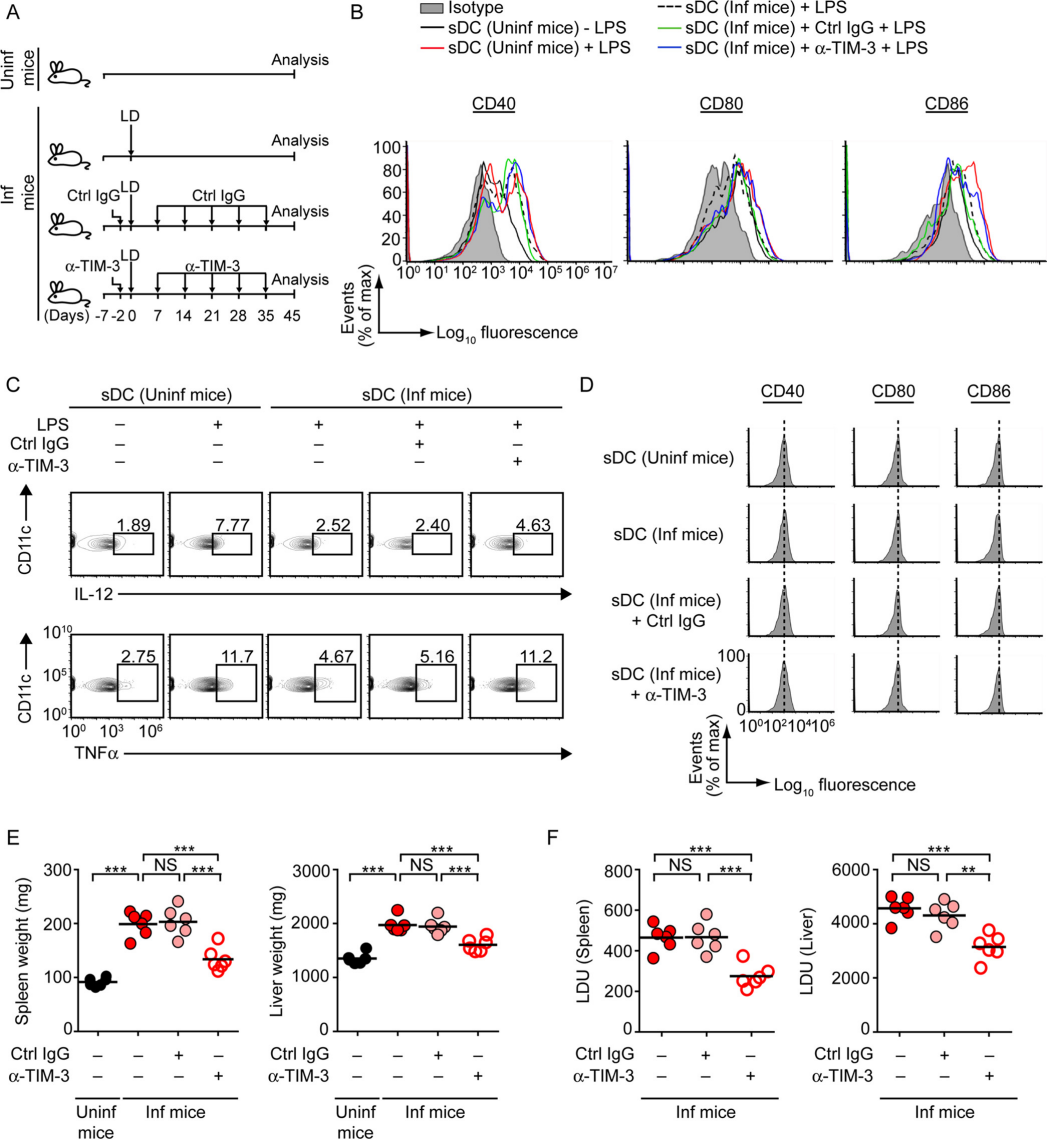
*In vivo* anti-TIM-3 antibody treatment prevents sDC suppression and reduces parasite load in L. donovani-infected mice. (A) Experimental procedure for anti-TIM-3 antibody treatment in mice. BALB/c mice were infected with L. donovani or left uninfected. In some experimental sets, L. donovani-infected mice were treated with control immunoglobulin (Ctrl IgG) or anti (α)-TIM-3 antibody 2 days prior to L. donovani infection and also on days 7, 14, 21, 28, and 35 following L. donovani infection. On the 45th day after L. donovani infection, livers and spleens were isolated for subsequent analyses. (B and C) Splenocytes isolated (on the 45th day postinfection) from uninfected or L. donovani-infected mice that had been treated (or not) with control immunoglobulin or anti-TIM-3 antibody (as in panel A) were treated with LPS for 24 h (+) or left untreated (−). The expression of costimulatory molecules on sDCs (B) and the frequencies of sDCs producing IL-12 and TNF-α (C) were analyzed via flow cytometry. sDCs (i.e., CD11c^+^ F4/80^−^ cells) were gated as in Fig. 3A. In panel C, numbers in outlined areas indicate the percentage of sDCs producing IL-12 or TNF-α. Results are representative of three separate analyses (see Fig. S4A and B for compiled data from three experiments). (D) Direct *ex vivo* analysis of costimulatory molecule expression on sDCs from mice infected with L. donovani or left uninfected and treated with control immunoglobulin or anti-TIM-3 antibody as described in panel A and analyzed via flow cytometry on day 45 postinfection. Results are representative of three different experiments (see Fig. S4C for combined mean fluorescence intensity data from three experiments). (E and F) Graphs showing the spleen and liver weights (E) and the parasite load (expressed as Leishman-Donovan units [LDU]) (F) of the above-described treated mice (as in panel A) analyzed on the 45th day after L. donovani infection. Combined data for two separate experiments (*n *= 3 mice per group in each experiment) are shown here. Each symbol denotes data for an individual mouse; bars indicate means. ***, *P* < 0.001; **, *P* < 0.01; NS, not significant.

Subsequently, we asked whether TIM-3 has any link with sDC suppression induced by L. donovani *in vivo*. For this, we treated BALB/c mice with a blocking anti-TIM-3 antibody (25) or isotype control antibody (i.e., control immunoglobulin [Ctrl IgG]) 2 days before L. donovani infection and also on days 7, 14, 21, 28, and 35 postinfection. In some experimental sets, we left BALB/c mice uninfected and untreated. On day 45 postinfection, we analyzed (via flow cytometry) the expression of costimulatory molecules and proinflammatory cytokines in sDCs following LPS treatment (Fig. 4A). Relative to sDCs from uninfected mice, sDCs of L. donovani-infected mice showed lower expression of costimulatory molecules and proinflammatory cytokines despite LPS stimulation (Fig. 4B and C; Fig. S4A and B). This low level of LPS-stimulated costimulatory molecule and proinflammatory cytokine expression in sDCs remained unaffected by the treatment of L. donovani-infected mice with isotype control antibody (Fig. 4B and C; Fig. S4A and B). In marked contrast, LPS stimulation efficiently increased the expression of costimulatory molecules and proinflammatory cytokines in sDCs derived from L. donovani-infected mice treated with anti-TIM-3 antibody (Fig. 4B and C; Fig. S4A and B). Notably, a direct *ex vivo* analysis of sDCs from uninfected and L. donovani-infected mice showed comparable expression profiles of costimulatory molecules (Fig. 4D; Fig. S4C). Furthermore, the expression level of costimulatory molecules on sDCs remained unaltered in L. donovani-infected mice despite *in vivo* anti-TIM-3 antibody treatment (Fig. 4D; Fig. S4C). Thus, our results have shown that the *in vivo* TIM-3 blockade with anti-TIM-3 antibody improves the responsiveness of sDCs of L. donovani-infected mice to subsequent DC maturation stimulus (e.g., LPS). These observations together support the hypothesis that TIM-3 is required for *in vivo* DC suppression in L. donovani-infected mice. In this regard, it could be argued that the anti-TIM-3 antibody might have blocked TIM-3-mediated inhibition of other cells (e.g., T cells [20]) regulating DC function and thereby indirectly improved the activation/maturation ability of sDCs in L. donovani-infected mice. Although our current findings do not rule out this possibility, TIM-3 is reported to be abundantly expressed by various DC lineages including sDCs (24, 36, 37). Accordingly, it is quite logical to believe that when anti-TIM-3 antibody was administered to L. donovani-infected mice, at least a fraction of this antibody was bound to TIM-3 expressed on sDCs and thereby prevented L. donovani-induced inhibition of sDCs. Thus, we propose here that in the *in vivo* context, L. donovani induces sDC suppression partly via TIM-3 expressed on the sDC surface. Collectively, these findings indicate the importance of TIM-3 in suppression of DCs *in vivo* during L. donovani infection.

10.1128/mbio.03309-21.4 FIG S4 Supporting information for Fig. 4. (A and B) Related to Fig. 4B and C. Graphs showing combined data of three experiments for MFIs of costimulatory molecule expression by sDCs (i.e., CD11c^+^ F4/80^−^ gated cells) (A) and the percentage of IL-12^+^ or TNF-α^+^ sDCs (B) presented in Fig. 4B and C, respectively. For these experiments, splenocytes were isolated from uninfected (Uninf) mice and mice infected for 45 days with L. donovani (Inf) that were treated (or not) with control immunoglobulin or anti-TIM-3 antibody as described in Fig. 4A. Splenocytes were then stimulated with LPS (for 24 h) and analyzed via flow cytometry. The MFIs were measured as in Fig. S1A and presented here as fold change compared to control sDCs (i.e., sDCs that were left unstimulated after isolation from uninfected mice given no antibody treatment). The gating strategies were followed as in Fig. S3. Corresponding flow cytometry data have been presented in Fig. 4B and C. (C) Related to Fig. 4D. Splenocytes of uninfected and 45-day-infected mice, treated or not with control immunoglobulin or anti-TIM-3 antibody as in Fig. 4A, were directly used to analyze the expression of costimulatory molecules on sDCs via flow cytometry. The respective flow cytometry data have been shown in Fig. 4D. Here, the relative MFI data (measured as in Fig. S1A) of costimulatory molecule expression on sDCs pooled from three separate analyses are plotted as bar graphs. The gating strategies were followed as described in Fig. S3A. In all panels, error bars indicate SD, and each symbol in the graphs corresponds to data derived from one independent experiment. ***, *P* < 0.001; **, *P* < 0.01; *, *P* < 0.05; NS, not significant. Download FIG S4, TIF file, 1.0 MB. Copyright © 2022 Akhtar et al. 2022 Akhtar et al. https://creativecommons.org/licenses/by/4.0/ This content is distributed under the terms of the Creative Commons Attribution 4.0 International license.

We then tested whether anti-TIM-3 antibody treatment at all can influence the parasite load in L. donovani-infected mice. Accordingly, we treated (or not) L. donovani-infected BALB/c mice with control antibody or anti-TIM-3 antibody as described above (Fig. 4A) and measured the spleen and liver weights and parasite loads on day 45 postinfection. We observed that treatment with anti-TIM-3 antibody, but not control antibody, considerably reduced the spleen and liver weights and parasite loads in L. donovani-infected mice (Fig. 4E and F). These results therefore suggest that blockade of TIM-3 with anti-TIM-3 antibody confers protection against L. donovani infection. Additionally, the findings of this experiment indicate a role for TIM-3 in disease pathogenesis by inhibiting antileishmanial immunity.

### DC-specific TIM-3 perturbs antileishmanial immune responses.

**FIG 5:**
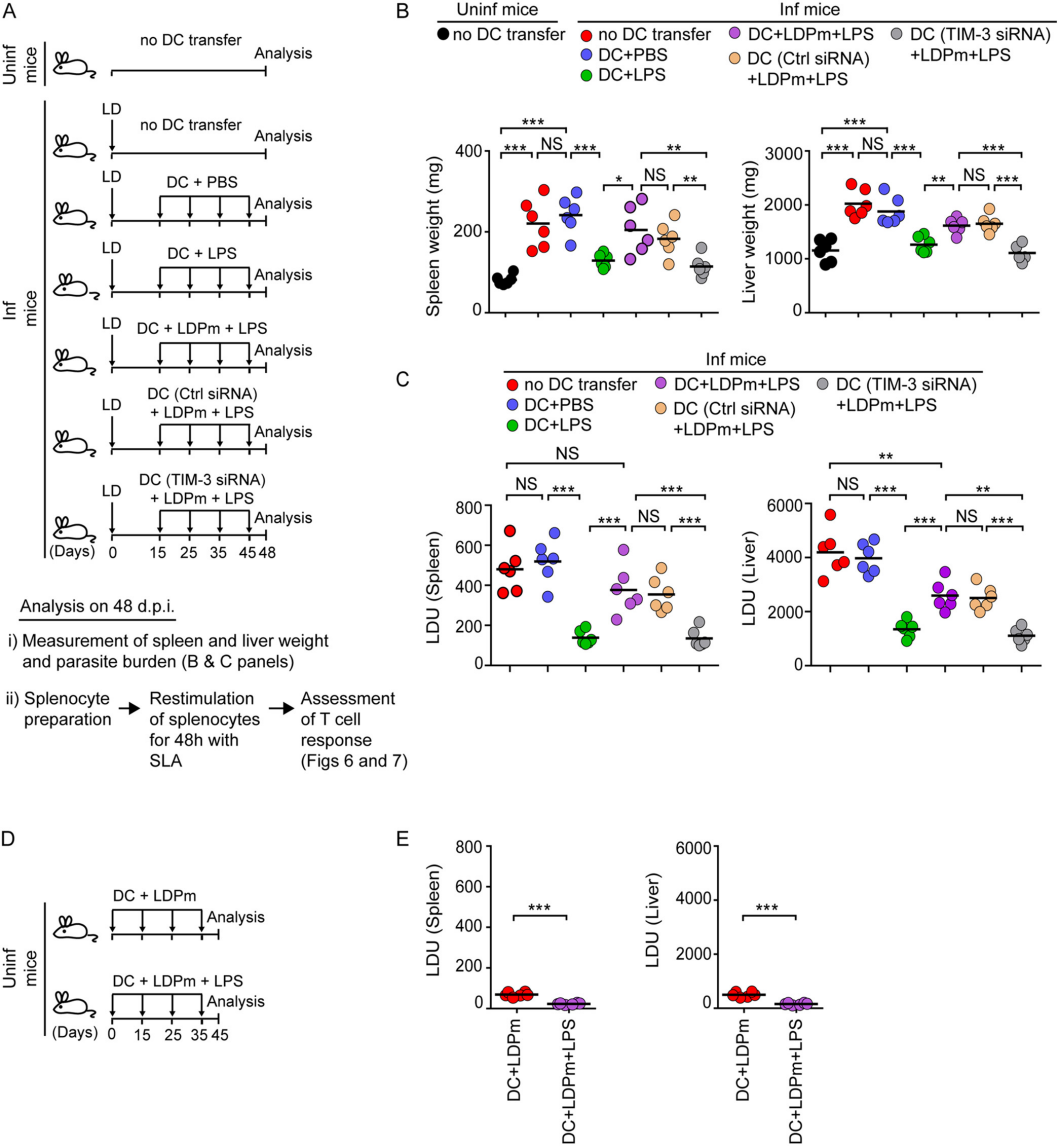
Suppression of TIM-3 expression improves the capacity of DCs to clear L. donovani infection in mice. (A) Schematic of DC adoptive transfer experiment. BALB/c BMDCs (1 × 10^6^) were either left untransfected or transfected with control siRNA or TIM-3-specific siRNA and then infected (or not) with LDPm for 24 h and treated with LPS for 24 h. In some experimental sets, BMDCs that had been kept untransfected and uninfected were treated for 24 h with PBS. Afterward, DCs (1 × 10^6^) were transferred intravenously into L. donovani-infected BALB/c mice (Inf mice) on indicated days after L. donovani infection. On the 48th day (after L. donovani infection), livers and spleens were isolated from these mice for measuring spleen and liver weight and parasite load (B and C, respectively). For some other experiments (Fig. 6 and 7), splenocytes were restimulated with soluble L. donovani antigens (SLA) for 48 h and then L. donovani-specific T cell responses were examined. (B and C) Graphs displaying spleen and liver weights (B) and the parasite load in the spleen and liver (C) (as in Fig. 4F) of the above-described treated mice, analyzed on the 48th day after L. donovani infection. (D) Experimental schematic for adoptive transfer of DCs to uninfected mice. BALB/c BMDCs (1 × 10^6^) were infected with LDPm for 24 h and then treated (or not) with LPS for another 24 h. DCs (1 × 10^6^) were then transferred into uninfected BALB/c mice on the indicated days. On the 45th day after the first DC transfer, spleens and livers were isolated for analysis. (E) Graphs showing splenic and liver parasite burden (analyzed as described for Fig. 4F) in uninfected mice treated as in panel D. Data in panels B, C, and E show combined results of two separate experiments (*n *= 3 mice per group in each experiment). Each symbol represents the data for an individual mouse; bars indicate means. ***, *P* < 0.001; **, *P* < 0.01; *, *P* < 0.05; NS, not significant.

Next, we assessed the ability of DC-expressed TIM-3 to influence antileishmanial T cell responses and its eventual impact on parasite load. This is because of the prevailing notion that DCs play a vital role in driving *Leishmania*-specific T cell reactivity (11). To investigate this scenario, we silenced (or not) TIM-3 expression in DCs by siRNA, infected these DCs with LDPm, and treated them with LPS. In some cases, we treated uninfected DCs with LPS or phosphate-buffered saline (PBS). After that, we transferred the above-described treated DCs into L. donovani-infected mice on days 15, 25, 35, and 45 following L. donovani infection (Fig. 5A). At 48 days after L. donovani infection, we measured spleen and liver weights (Fig. 5B) and the parasite burden in these organs (Fig. 5C). Consistent with a previous report (38), adoptive transfer of LPS-treated DCs markedly reduced the liver and spleen weights and parasite load in L. donovani-infected mice (Fig. 5B and C). Such inhibitory effects of LPS-treated DCs were greatly compromised when we infected these DCs with LDPm before LPS stimulation (Fig. 5B and C). However, suppression of TIM-3 expression restored the above-mentioned inhibitory ability of LPS-treated DCs despite LDPm infection (Fig. 5B and C). Now, here a question could be raised that the excess parasite burden in L. donovani-infected mice receiving LPS-treated LDPm-infected DCs compared to those receiving LPS-treated uninfected DCs (Fig. 5C; violet versus green symbols) might be contributed by the infected DCs themselves. To verify this possibility, we performed another adoptive transfer experiment wherein we infected BMDCs with LDPm for 24 h. We then treated these BMDCs with LPS for an additional 24 h or left them untreated and transferred these cells into uninfected mice on days 0, 15, 25, and 35. On the 45th day, we analyzed the liver and splenic parasite load (Fig. 5D). Whereas transfer of LDPm-infected DCs somewhat increased the splenic and liver parasite load (splenic and liver Leishman-Donovan unit [LDU] counts were 69.23 ± 11.12 and 503.50 ± 92, respectively) in uninfected mice, only few parasites were detected in the spleens and livers of uninfected mice following the transfer of LPS-stimulated LDPm-infected DCs (splenic and liver LDU counts were 23.08 ± 3.9 and 159.83 ± 37.85, respectively) (Fig. 5E). In fact, consistent with a prior report (39), LPS treatment for 24 h considerably reduced the percentage of infected DCs and the number of intracellular parasites within LDPm-infected DCs (Fig. S5A), which were subsequently used for adoptive transfer experiments described in Fig. 5A. Therefore, the intracellular parasites within LPS-stimulated LDPm-infected DCs might have a minimal effect in elevating the splenic and liver parasite burden in L. donovani-infected mice following adoptive transfer.

10.1128/mbio.03309-21.5 FIG S5 Assessment of L. donovani infection in DCs. (A) BMDCs (1 × 10^6^) were infected for 24 h with LDPm at a parasite-to-DC ratio of 10:1. Afterward, BMDCs were treated with LPS for 24 h. The percentage of infected BMDCs and the number of intracellular amastigotes/1,000 BMDCs before and after LPS treatment were determined by Giemsa staining and are presented here as bar graphs. Data shown are a compilation of two separate experiments (*n *= 3 in each experiment). (B) BMDCs were infected with LDPm (as in panel A) for 6 h, 12 h, and 24 h. The percentage of infected BMDCs (enumerated by Giemsa staining) is shown here graphically. In all panels, error bars indicate SD, and each symbol in panels A and B represents data of individual replicate or one independent experiment, respectively. ***, *P* < 0.001. Download FIG S5, TIF file, 0.3 MB. Copyright © 2022 Akhtar et al. 2022 Akhtar et al. https://creativecommons.org/licenses/by/4.0/ This content is distributed under the terms of the Creative Commons Attribution 4.0 International license.

**FIG 6:**
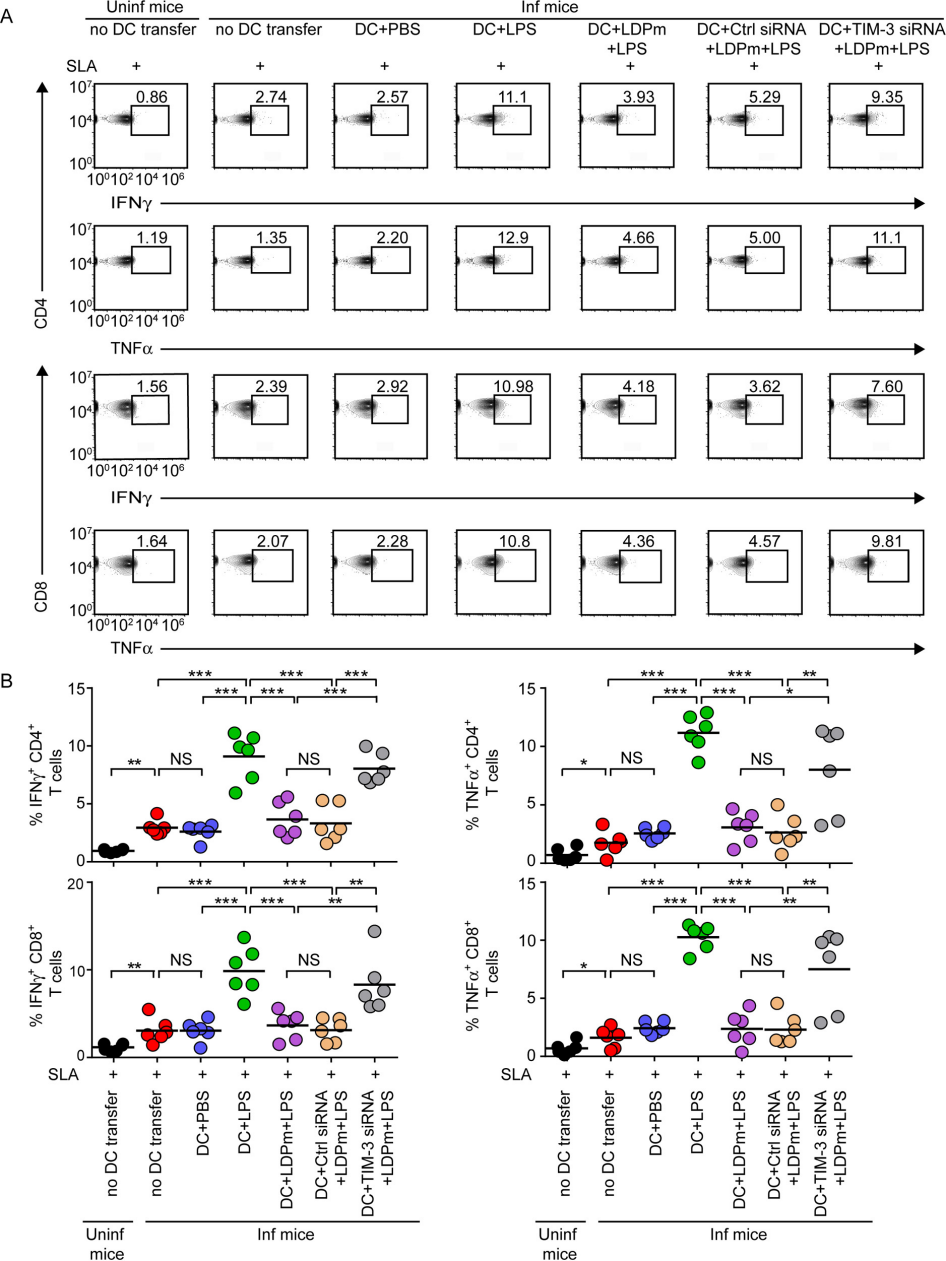
TIM-3 on DCs impedes type 1 T cell responses in L. donovani-infected mice. TIM-3-silenced BMDCs and control-silenced BMDCs (1 × 10^6^) were infected for 24 h with LDPm and then treated with LPS for 24 h and adoptively transferred into L. donovani-infected mice (experimental setup given in Fig. 5A). On the 48th day postinfection, splenocytes (1 × 10^6^/well) prepared from these mice were restimulated with SLA for 48 h and then assays for antigen-specific T cell responses (see Fig. S7 for antigen specificity) were performed. (A) Frequencies of IFN-γ- and TNF-α-expressing CD4^+^ or CD8^+^ T cells in SLA-stimulated splenocytes of the above-mentioned mice were enumerated via flow cytometry (representative data for *n *= 6). Numbers in the outlined areas indicate the percentage of CD4^+^ or CD8^+^ T cells producing IFN-γ or TNF-α. The gating strategy for flow cytometry analysis is mentioned in Fig. S6A and B, and the antigen specificity of this assay has been shown in Fig. S7A and B. (B) Graphs depict (compiled data for two experiments; *n *= 3 mice per group in each experiment) the percentage of CD4^+^ and CD8^+^ T cells producing IFN-γ or TNF-α. Each symbol represents the data for an individual mouse, and bars indicate means. ***, *P* < 0.001; **, *P* < 0.01; *, *P* < 0.05; NS, not significant.

**FIG 7:**
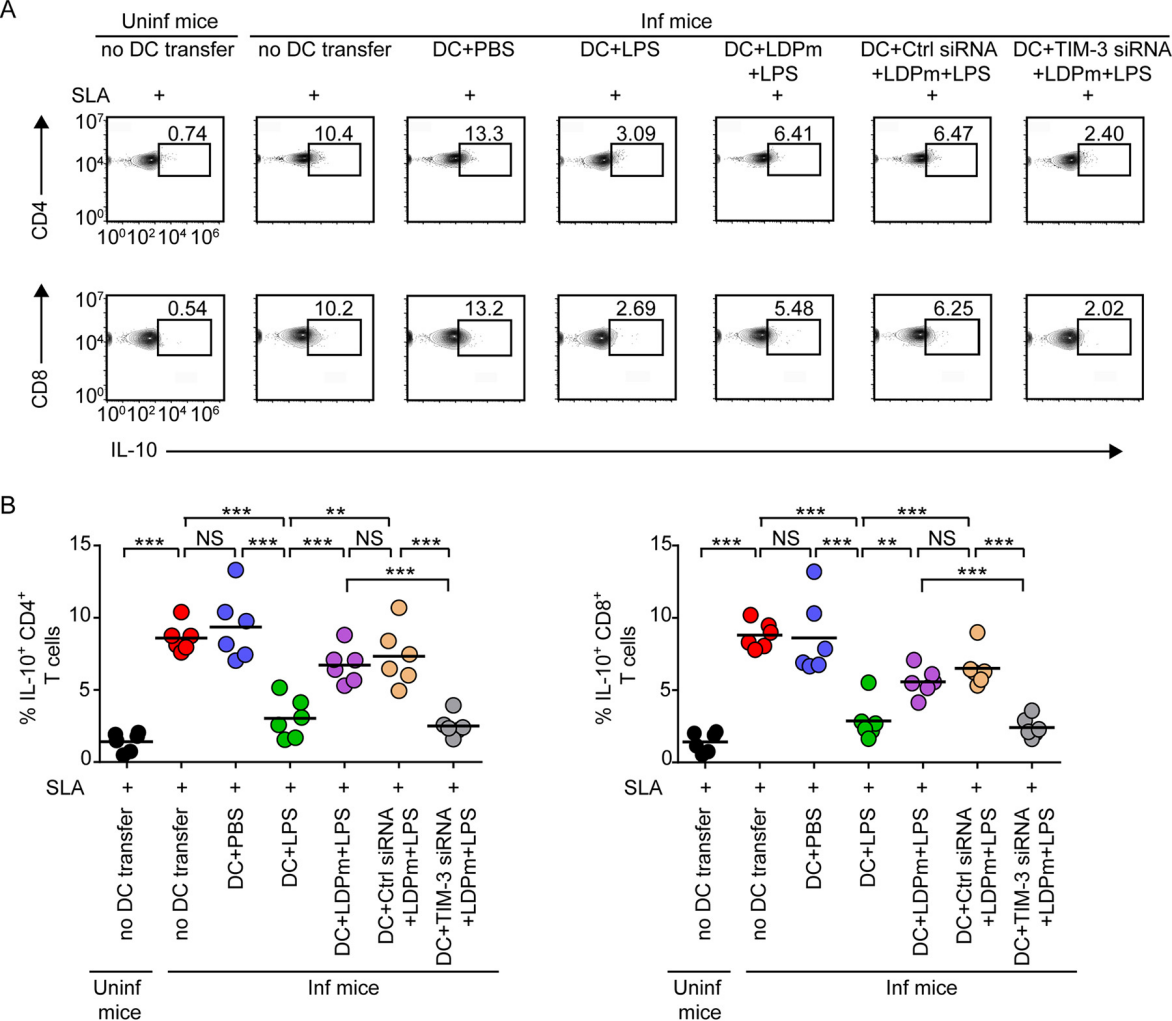
TIM-3 expressed by DCs promotes type 2 T cell responses in L. donovani-infected mice. (A) Percentage of IL-10-expressing CD4^+^ or CD8^+^ T cells detected within SLA-restimulated splenocytes of uninfected and L. donovani-infected mice following adoptive transfer of BMDCs that were control silenced or TIM-3 silenced, infected with LDPm for 24 h, subsequently treated for 24 h with LPS (Fig. 5 and 6 give experimental details), and analyzed via flow cytometry (a representative result of *n *= 6). Numbers in the outlined areas indicate the percentage of IL-10-expressing CD4^+^ and CD8^+^ T cells. See Fig. S6C for the gating strategy and Fig. S7A and C for the antigen specificity of this assay. (B) Graphs showing (compiled data for two individual experiments; *n *= 3 mice per group in each experiment) the percentage of CD4^+^ or CD8^+^ T cells expressing IL-10. Each symbol corresponds to an individual mouse, and bars indicate means. ***, *P* < 0.001; **, *P* < 0.01; NS, not significant.

We then examined the type 1 and type 2 T cell responses in L. donovani-infected mice following adoptive transfer of DCs treated as described in Fig. 5A. For this purpose, we prepared splenocytes from the above-described treated mice on the 48th day postinfection, restimulated these cells with soluble L. donovani antigens (SLA) for 48 h (40, 41), and assessed the frequencies of type 1 (interferon gamma [IFN-γ] and TNF-α) and type 2 (IL-10) cytokine-producing CD4^+^ or CD8^+^ T cells via intracellular staining, followed by flow cytometry. We found that the frequencies of CD4^+^ and CD8^+^ T cells expressing IFN-γ and TNF-α were increased and those expressing IL-10 were decreased (also noted by another group [38]) in the spleens of L. donovani-infected mice following adoptive transfer of LPS-treated but not control (PBS)-treated DCs (Fig. 6A and B, Fig. 7A and B, and Fig. S6). Such alteration of IFN-γ-, TNF-α-, or IL-10-expressing CD4^+^ or CD8^+^ T cell populations was largely impeded when we infected LPS-treated DCs with LDPm before adoptive transfer (Fig. 6A and B, Fig. 7A and B, and Fig. S6). In contrast, silencing of TIM-3 expression endowed these LDPm-infected LPS-treated DCs with the capacity to increase IFN-γ- and TNF-α-expressing CD4^+^ and CD8^+^ T lymphocyte populations and reduce IL-10-expressing CD4^+^ and CD8^+^ T lymphocyte populations in L. donovani-infected mice (Fig. 6A and B, Fig. 7A and B, and Fig. S6). Notably, the antigen specificity of this assay is shown in Fig. S7. Our data demonstrated that the frequency of IFN-γ- and TNF-α-producing CD4^+^ or CD8^+^ T cells was increased in uninfected mice following adoptive transfer of LPS-treated DCs and that these frequencies remained unchanged despite *in vitro* restimulation of spleen cells with soluble L. donovani antigens (SLA) (Fig. S7A and B). Compared to uninfected mice, L. donovani-infected mice showed more IFN-γ- and TNF-α-producing T cells (in the absence of antigenic stimulation of spleen cells) upon transfer of LPS-stimulated DCs (Fig. S7A and B). This increase in the IFN-γ- and TNF-α-producing T cell population in L. donovani-infected mice upon LPS-stimulated DC transfer was markedly augmented when we restimulated the spleen cells of these mice with SLA (Fig. S7A and B). We also observed that without any DC transfer, L. donovani-infected mice showed more IL-10-expressing T cells than uninfected mice (Fig. S7C). In addition, restimulation with SLA *in vitro* further increased the frequency of IL-10-expressing T cells derived from L. donovani-infected mice but not of those derived from uninfected mice (Fig. S7C). These results together confirm that DCs regulate antileishmanial T cell responses via TIM-3 in an antigen-specific manner. Overall, our findings suggest that L. donovani utilizes TIM-3 of DCs to dampen antileishmanial immunity.

10.1128/mbio.03309-21.6 FIG S6 Gating strategies for Fig. 6 and 7. Initially, lymphocytes were gated on the basis of forward scatter, side scatter, and CD3 expression. The CD3^+^ lymphocytes were further gated into CD4^+^ and CD8^+^ fractions. Then, the percentage of type 1 (IFN-γ [A] and TNF-α [B]) or type 2 (IL-10 [C]) cytokine-expressing cells within the CD4^+^ and CD8^+^ gated T cell population was analyzed based on background staining with respective isotype control antibodies. Download FIG S6, TIF file, 1.0 MB. Copyright © 2022 Akhtar et al. 2022 Akhtar et al. https://creativecommons.org/licenses/by/4.0/ This content is distributed under the terms of the Creative Commons Attribution 4.0 International license.

10.1128/mbio.03309-21.7 FIG S7 Assessment of antigen specificity for the assays in Fig. 6 and 7. (A) Schematic of DC adoptive transfer experiments to determine antigen specificity of T cell responses. BALB/c BMDCs (1 × 10^6^) were treated with PBS or LPS for 24 h and then adoptively transferred to L. donovani-infected BALB/c mice on indicated days postinfection. In some experimental sets, BMDCs treated as described above were similarly transferred to age-matched uninfected BALB/c mice. Alternatively, both uninfected and L. donovani-infected mice were left without any DC transfer. On the 48th day postinfection, splenocytes isolated from these mice were restimulated with SLA for 48 h or left unstimulated. The antigen-specific T cell responses were then evaluated (via flow cytometry) by analyzing the changes in frequency of type 1 and type 2 cytokine-expressing CD4^+^ or CD8^+^ T cells (described below) following SLA-mediated restimulation. (B and C) Graphs depict the frequencies of CD4^+^ or CD8^+^ T cells expressing type 1 cytokines (IFN-γ and TNF-α [B]) or type 2 cytokine (IL-10 [C]; analyzed via flow cytometry) after restimulation (or without restimulation) of splenocytes from above-mentioned mice with SLA. Compiled data of two experiments (*n *= 3 mice per group in each experiment) are presented here. Each symbol represents the data of an individual mouse, and bars indicate means. ***, *P* < 0.001; **, *P* < 0.01; *, *P* < 0.05; NS, not significant. Download FIG S7, TIF file, 1.1 MB. Copyright © 2022 Akhtar et al. 2022 Akhtar et al. https://creativecommons.org/licenses/by/4.0/ This content is distributed under the terms of the Creative Commons Attribution 4.0 International license.

### L. donovani-induced DC suppression relies on TIM-3–Btk signaling.

**FIG 8:**
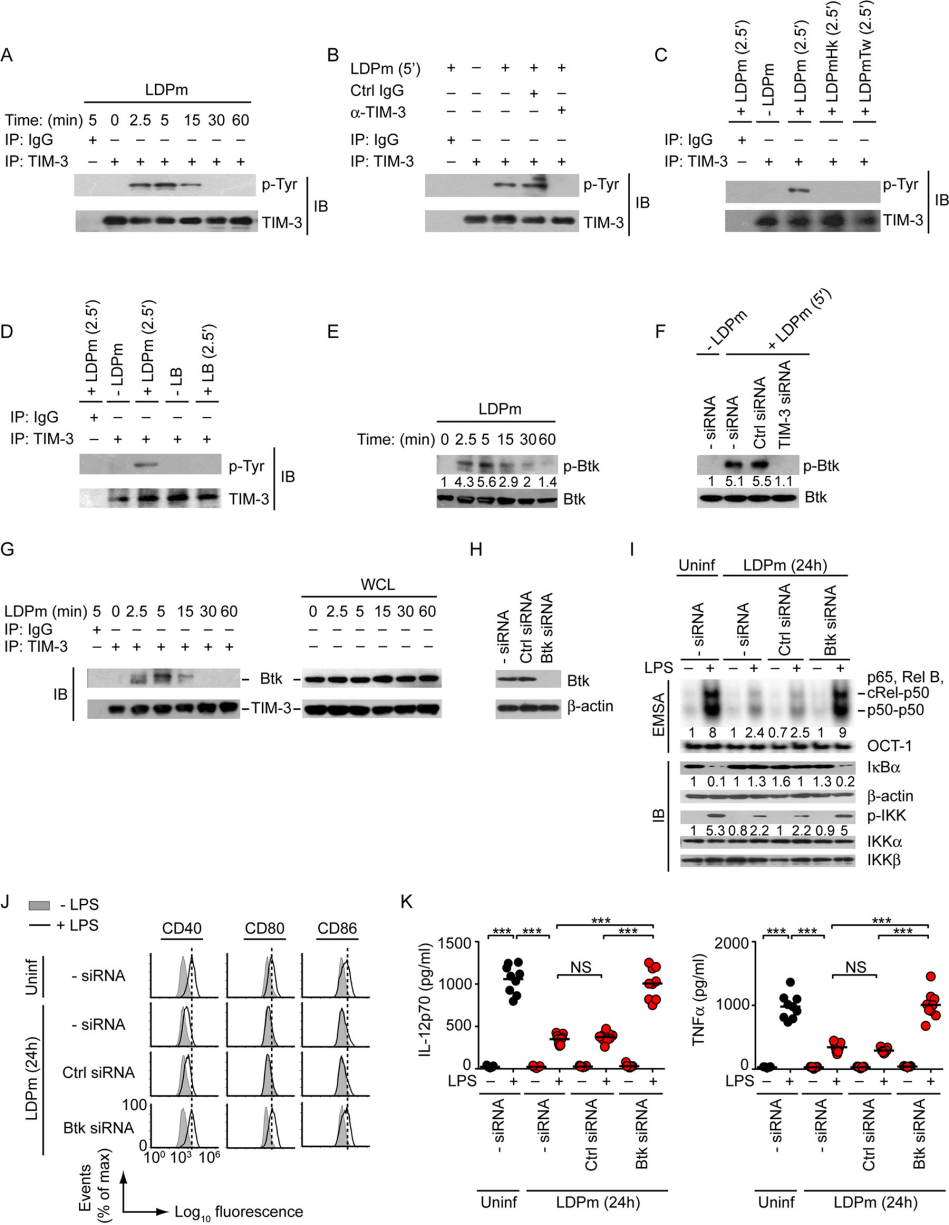
TIM-3–Btk signaling mediates L. donovani-induced DC suppression. (A) BMDCs were incubated with LDPm for the specified times. Cell lysates were immunoprecipitated (IP) with anti-TIM-3 antibody and immunoblotted for phosphorylated tyrosine (p-Tyr) or TIM-3. IgG, immunoglobulin G (IP control). (B) BMDCs were left unincubated (−) or incubated for 5 min with LDPm (+). In some experimental sets, BMDCs were treated with control immunoglobulin or anti-TIM-3 antibody for 1 h prior to incubation with LDPm. The Tyr phosphorylation of TIM-3 was examined as in panel A. (C) Analysis of Tyr phosphorylation of TIM-3 (as in panel A) in BMDCs that had been kept separated from LDPm by a transwell insert (LDPmTw) or cocultured with live (also represented as LDPm) or heat-killed (LDPmHk) LDPm for 2.5 min. (D) BMDCs were incubated (or not) with LDPm or polystyrene latex beads (LB) for 2.5 min. The Tyr phosphorylation of TIM-3 was examined as in panel A. (E) Expression of total and phosphorylated Btk in BMDCs incubated with LDPm for indicated times; assessed via immunoblotting. Numbers below lanes indicate densitometry of phosphorylated Btk, normalized to total Btk and presented relative to BMDCs incubated with LDPm for 0 min. (F) Effect of TIM-3 silencing on the expression of phosphorylated Btk in BMDCs incubated with LDPm for 5 min, determined by immunoblotting. Numbers below lanes indicate densitometry results (as in panel E), presented relative to BMDCs that were kept untransfected (− siRNA) and not incubated with LDPm (− LDPm). (G) BMDCs were cocultured with LDPm for various times (minutes, above lanes). Interaction between Btk and TIM-3 was assessed by immunoprecipitation, followed by immunoblot analysis. WCL is whole-cell lysate (no IP), and IgG indicates IP control. (H) Immunoblot analysis showing the efficiency of siRNA-mediated silencing of Btk in BMDCs, with β-actin as a loading control. (I) EMSA evaluating NF-κB or OCT-1 DNA-binding activity (top) and immunoblot analysis showing expression of indicated proteins (bottom) in BMDCs transfected as in panel H and then infected for 24 h with LDPm or left uninfected (Uninf) and cultured with or without LPS for 0.5 h. Numbers below lanes indicate relative densitometry results (as in Fig. 1F). (J and K) Flow cytometry analysis of costimulatory molecule expression (J) and ELISA of IL-12p70 and TNF-α secretion (K) by BMDCs that had been transfected as in panel H, infected for 24 h with LDPm or kept uninfected, and then cultured with or without LPS for 24 h. Data in panels A, B, and E to J are representative of three independent experiments, data in panels C and D are representative of two separate experiments, and data in panel K are a compilation of three separate experiments (*n *= 3 per experiment). In panel K, each symbol corresponds to data for an individual replicate, and bars indicate means. The densitometry results for panels E, F, and I and relative mean fluorescence intensities of costimulatory molecule expression for panel J pooled from three independent experiments are presented in Fig. S9. ***, *P* < 0.001; NS, not significant.

Having found that TIM-3 was necessary for L. donovani-induced inhibition of DCs and subsequent antileishmanial immune responses, we made an effort to identify the TIM-3 signaling events mediating this inhibitory effect of L. donovani. Initially, we tested whether L. donovani can trigger the TIM-3 receptor on DCs. To verify this possibility, we incubated BMDCs with LDPm for various times and then immunoprecipitated TIM-3 with anti-TIM-3 antibody and measured tyrosine (Tyr) phosphorylation via Western blotting (23). We found that BMDC incubation with LDPm led to an intense and rapid (within 2.5 to 5 min) increase in Tyr phosphorylation of TIM-3, albeit in a transient manner (Fig. 8A). Pretreatment of BMDCs with anti-TIM-3 antibody, however, blocked this effect (Fig. 8B). Notably, within 2.5 to 5 min of incubation with L. donovani parasites, approximately 20 to 27% of BMDCs (the average number of attached promastigotes per DC was 2.5 ± 0.06 at 2.5 min and 4.92 ± 0.35 at 5 min) had attached LDPm on their surface (Fig. S8). Our finding is consistent with an earlier report (42) demonstrating the adherence of a significant proportion (42 to 55%) of target host cells with *Leishmania* parasites within a 2- to 5-min incubation time. Aside from parasite adherence, a transient interaction between L. donovani and DCs (which might be nonproductive in causing successful parasite attachment to the DC surface) also possibly contributes in triggering TIM-3 phosphorylation in DCs. Further, it is noteworthy here that *Leishmania* parasites have been reported to trigger host cell signaling (e.g., Ca^2+^ signaling) within 1 min of incubation (43). In view of the latter report, the observed increase in TIM-3 phosphorylation within 2.5 min of incubation with L. donovani does not seem to be an unusual event. Thus, our data together with the above-mentioned report indicate that L. donovani can directly interact with DCs within this short time and that this L. donovani-DC interaction triggers TIM-3 phosphorylation. In fact, separation of LDPm from DCs by a cell-impermeable membrane abolished TIM-3 phosphorylation in DCs (Fig. 8C). Interestingly, only live but not heat-killed LDPm showed the ability to induce TIM-3 phosphorylation in DCs (Fig. 8C). Our data further demonstrated that the induction of TIM-3 phosphorylation in DCs was L. donovani specific. For example, incubation of DCs with polystyrene latex beads did not induce TIM-3 phosphorylation (Fig. 8D). Together, our results demonstrate the ability of L. donovani to activate the TIM-3 receptor on DCs. In addition, the above findings confirmed the TIM-3-inhibiting potential of anti-TIM-3 antibody used in this study.

10.1128/mbio.03309-21.8 FIG S8 Assessment of L. donovani parasite attachment to DCs. (A) LDPm and BMDCs were labeled with CFSE and eFluor 670, respectively, and then cocultured in 24-well plates for 2.5 and 5 min (details given in Materials and Methods). The percentage of DCs with attached LDPm (i.e., CFSE^+^ eFluor 670^+^ population [in red-outlined areas]) was enumerated via flow cytometry. (B) The number of LDPm adhering to individual BMDCs at 2.5 min or 5 min after LDPm incubation was determined by Giemsa staining and is plotted here as bar graphs (left panel). Right panel, representative image showing parasite attachment to DCs (indicated by arrows). Data in panel A are representative of two independent experiments, and data shown in panel B are a compilation of two separate experiments (*n *= 2 per experiment). In panel B, error bars indicate SD and each symbol corresponds to the data of an individual replicate. Download FIG S8, TIF file, 1.7 MB. Copyright © 2022 Akhtar et al. 2022 Akhtar et al. https://creativecommons.org/licenses/by/4.0/ This content is distributed under the terms of the Creative Commons Attribution 4.0 International license.

10.1128/mbio.03309-21.9 FIG S9 Supporting information for Fig. 8. (A) Related to Fig. 8E. Compiled densitometry data from three separate experiments for immunoblot analysis showing phosphorylated Btk expression in BMDCs incubated with LDPm for indicated times (presented in Fig. 8E) are plotted as a bar graph. Densitometry quantification was done as indicated in Fig. 8E and presented relative to BMDCs incubated with LDPm for 0 min. (B) Related to Fig. 8F. The bar graph shows densitometry data (combined from three separate experiments) for Fig. 8F assessing the expression of phosphorylated Btk in BMDCs left untransfected or transfected with specified siRNAs and then incubated for 5 min with LDPm or left unincubated. Densitometry quantification was done as illustrated in Fig. 8E and is presented relative to BMDCs that had been left untransfected (− siRNA) and unincubated (− LDPm). (C) Related to Fig. 8I. Graphical presentation of densitometry data pooled from three separate experiments measuring the intensity of NF-κB DNA binding and the expression of IκBα and phosphorylated IKK in BMDCs transfected with specified siRNAs, infected for 24 h with LDPm or left uninfected, and then cultured with or without LPS. Densitometry quantification was done as in Fig. 8I and presented as in Fig. 8I. (D) Related to Fig. 8J. Bar graphs show combined results of three individual analyses for MFI values of costimulatory molecule expression (shown in Fig. 8J) on BMDCs transfected with indicated siRNAs, infected with LDPm for 24 h or left uninfected, and then treated with LPS for an additional 24 h. The MFI values were measured as mentioned in Fig. S1A and presented as fold change compared to BMDCs kept untransfected and uninfected and given no LPS treatment. In all panels, error bars denote SD and each symbol in the graphs corresponds to data derived from one independent experiment. ***, *P* < 0.001; **, *P* < 0.01; *, *P* < 0.05; NS, not significant. Download FIG S9, TIF file, 0.8 MB. Copyright © 2022 Akhtar et al. 2022 Akhtar et al. https://creativecommons.org/licenses/by/4.0/ This content is distributed under the terms of the Creative Commons Attribution 4.0 International license.

We then searched for the signaling mediator, which acted downstream of TIM-3. Previously, we have reported that Bruton’s tyrosine kinase (Btk), a Tec-family nonreceptor tyrosine kinase, serves as a proximal signal element of the TIM-3 signaling pathway and plays a key role in DC suppression (23). Accordingly, we examined whether triggering of TIM-3 by L. donovani activates Btk in DCs. We analyzed Btk activation by measuring phosphorylation of the Btk Tyr223 via Western blotting (23). At 2.5 to 5 min after incubation with LDPm, Btk phosphorylation was upregulated in BMDCs (Fig. 8E; Fig. S9A). However, silencing of TIM-3 expression blocked this effect (Fig. 8F; Fig. S9B). Coimmunoprecipitation analyses further showed that within 2.5 to 5 min postincubation, LDPm induced physical interaction between Btk and TIM-3 (Fig. 8G). These findings demonstrate that Btk participates in L. donovani-induced TIM-3 signaling in DCs.

To test a functional role of Btk in L. donovani-mediated suppression of the NF-κB pathway and DC activation/maturation, we silenced Btk expression in BMDCs by siRNA (Fig. 8H). We found that silencing of Btk blocked the inhibitory effect of LDPm on LPS-stimulated activation of NF-κB signaling, upregulation of costimulatory molecules, and secretion of IL-12 and TNF-α by DCs (Fig. 8I to K; Fig. S9C and D). Together, these data demonstrate that TIM-3–Btk acts as a key signaling axis through which L. donovani inhibits NF-κB signaling and eventually suppresses DCs.

### TIM-3–Btk signaling drives L. donovani-induced DC suppression in an IL-10-dependent manner.

**FIG 9:**
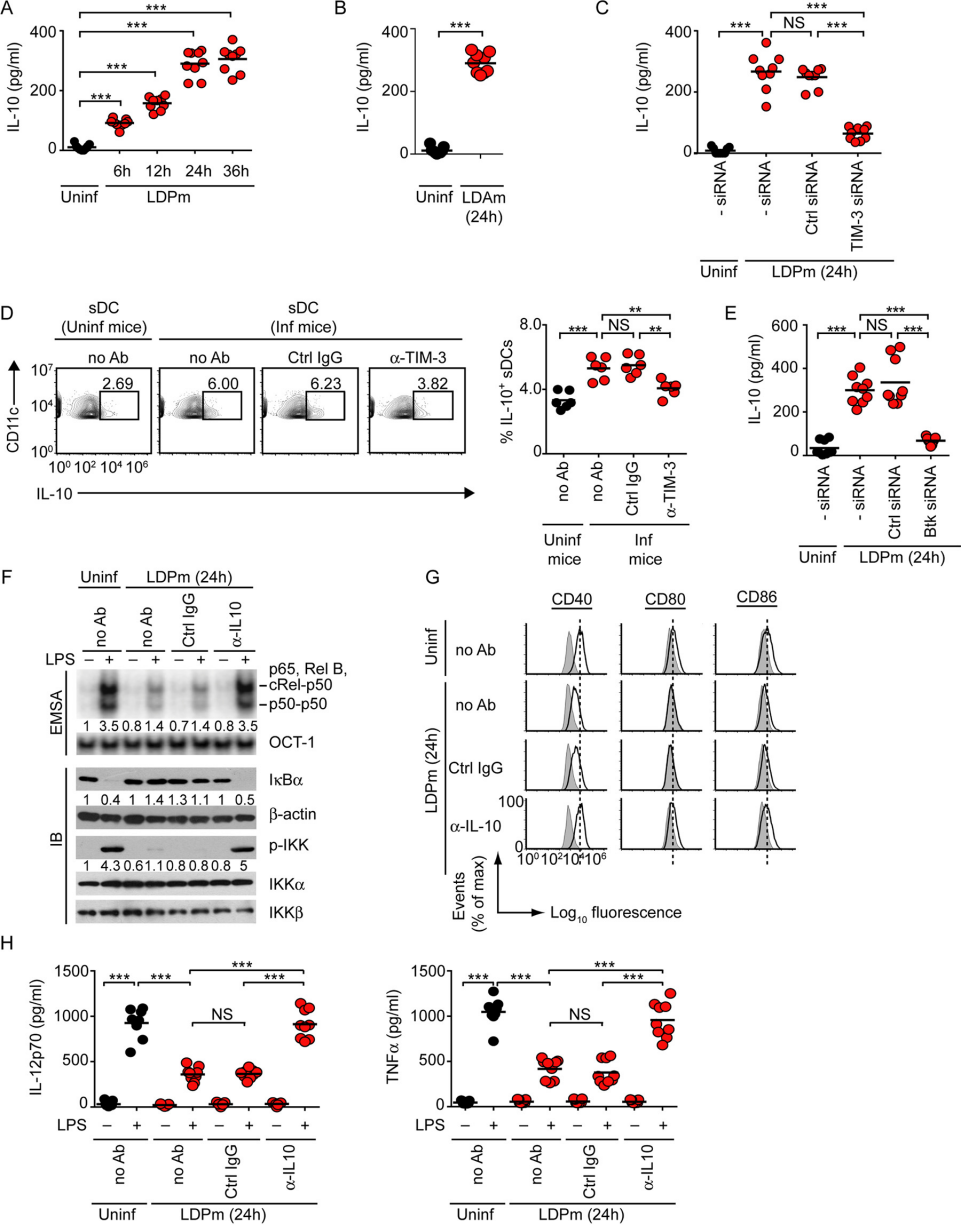
The TIM-3–Btk signaling promotes L. donovani-induced DC suppression via IL-10. (A to C) BMDCs were either infected with LDPm (A) or LDAm (B) for specified times or kept uninfected. Alternatively, BMDCs were treated with indicated siRNAs prior to LDPm infection (C). IL-10 secretion from BMDCs was analyzed via ELISA. Data are combined from three experiments with 3 replicates per experiment. Each symbol corresponds to data for an individual replicate, and bars indicate means. (D) Representative (*n *= 6) flow cytometry analysis showing the frequency of IL-10-expressing sDCs (i.e., CD11c^+^ F4/80^−^ gated cells; see Fig. S10A for gating strategy) in splenocytes of uninfected and L. donovani-infected mice treated with no antibody (no Ab), control immunoglobulin, or anti-TIM-3 antibody as described in Fig. 4A. Numbers represent the percentage of IL-10-expressing sDCs in the outlined areas (left panel). Right panel: graph shows corresponding data combined from two separate experiments with 3 mice per group in each experiment. Each symbol indicates data for an individual mouse, and bars indicate means. (E) Effect of Btk silencing on IL-10 secretion by BMDCs infected for 24 h with LDPm and analyzed by ELISA. Combined data for three individual experiments (*n *= 3 in each experiment) are shown. Each symbol corresponds to data of an individual replicate, and bars indicate means. (F) BMDCs were left uninfected (Uninf) or infected for 24 h with LDPm in the absence (no Ab) or presence of control immunoglobulin or anti-IL-10 antibody and then treated with or without LPS for 0.5 h. NF-κB or OCT-1 DNA binding was assessed by EMSA, and the expression of indicated proteins was analyzed via immunoblotting. Numbers below lanes indicate relative densitometry quantification (as in Fig. 1F). Results are representative of three separate analyses. The summary of densitometry results of three individual experiments is presented in Fig. S10B. (G and H) BMDCs were kept uninfected or infected with LDPm (for 24 h) in the presence of indicated antibodies and then cultured for 24 h with or without LPS. Expression of costimulatory molecules was analyzed via flow cytometry (G), and secretion of IL-12p70 and TNF-α was quantified by ELISA (H). One representative set of flow cytometry data (out of three experiments) is shown in panel G, and the relative mean fluorescence intensity data pooled from three separate experiments are presented in Fig. S10C. The ELISA data shown in panel H are a compilation of three different experiments (*n *= 3 in each experiment). Each symbol denotes data for an individual replicate; bars indicate means. ***, *P* < 0.001; **, *P* < 0.01; NS, not significant.

Next, to determine how L. donovani-induced TIM-3–Btk signaling suppressed the activation and maturation of DCs, we examined a role for IL-10 in this process. This is because of the fact that IL-10 is known to inhibit DCs (44). Earlier, we and others have demonstrated that IL-10 is produced by DCs during L. donovani infection (9, 45). However, it is still unclear whether L. donovani utilizes IL-10 to inhibit DCs and how L. donovani induces IL-10 secretion from DCs. In addition, it has remained specifically undefined whether TIM-3 and Btk play any role in regulating IL-10 production in response to L. donovani infection. To address all these issues, we first analyzed whether L. donovani induces IL-10 production by BMDCs. Temporal analyses showed that IL-10 secretion from BMDCs was augmented as early as 6 h after LDPm infection and reached maximum at 24 to 36 h postinfection (Fig. 9A). Similar to LDPm, LDAm triggered IL-10 secretion from BMDCs (Fig. 9B). Silencing of TIM-3 expression, on the other hand, attenuated L. donovani-induced IL-10 secretion from BMDCs (Fig. 9C). Notably, the IL-10-expressing sDCs were found to be more abundant in L. donovani-infected mice than in uninfected mice (Fig. 9D; Fig. S10A). However, the frequency of IL-10-expressing sDCs was substantially reduced when we treated L. donovani-infected mice with anti-TIM-3 antibody (Fig. 9D; Fig. S10A). We further observed that similar to TIM-3 silencing (Fig. 9C), Btk silencing prevented the increased IL-10 production by BMDCs despite LDPm infection (Fig. 9E). Thus, TIM-3 and Btk play a crucial role in L. donovani-induced IL-10 secretion from DCs.

10.1128/mbio.03309-21.10 FIG S10 Supporting information for Fig. 9. (A) Gating strategy related to Fig. 9D. The CD11c^+^ F4/80^−^ cells within splenocytes were gated as indicated. The gated CD11c^+^ F4/80^−^ cells were then examined for IL-10^+^ population (Fig. 9D) after exclusion of background signal (isotype control). (B) Related to Fig. 9F. Bar graphs show a combined densitometry quantification (from three separate experiments) for Fig. 9F depicting NF-κB DNA binding and the expression of IκBα and phosphorylated IKK in BMDCs that were infected (for 24 h) with LDPm in the absence (no Ab) or presence of indicated antibodies and then stimulated with LPS for 0.5 h (+) or left unstimulated (−). Uninf, uninfected BMDCs. Densitometry quantification was performed as mentioned in Fig. 1F and presented relative to uninfected BMDCs cultured in the absence of antibody (no Ab) and subsequently left unstimulated. (C) Related to Fig. 9G. Bar graphs depict the combined MFI values of costimulatory molecule expression (shown in Fig. 9G) from three experiments. BMDCs were infected (for 24 h) with LDPm in the presence of indicated antibodies and then stimulated with LPS for 24 h or left unstimulated. The MFI values were measured as described in Fig. S1A and are presented as fold change compared to uninfected BMDCs cultured in the absence of antibody (no Ab) and then kept unstimulated. Error bars represent SD. Each symbol in the graphs (in panels B and C) corresponds to data derived from one independent experiment. ***, *P* < 0.001; **, *P* < 0.01; *, *P* < 0.05; NS, not significant. Download FIG S10, TIF file, 0.9 MB. Copyright © 2022 Akhtar et al. 2022 Akhtar et al. https://creativecommons.org/licenses/by/4.0/ This content is distributed under the terms of the Creative Commons Attribution 4.0 International license.

Subsequently, to assess whether L. donovani-mediated induction of IL-10 production had any relevance in DC suppression, we monitored the effect of IL-10 neutralization with an anti-IL-10 antibody. Whereas LDPm effectively inhibited LPS-stimulated NF-κB signaling, upregulation of costimulatory molecule expression, and secretion of IL-12 and TNF-α by BMDCs treated with isotype control antibody (control immunoglobulin [Ctrl IgG]), anti-IL-10 neutralizing antibody blocked these effects (Fig. 9F to H; Fig. S10B and C). Together, these results revealed that TIM-3–Btk acts as a critical signaling element in driving L. donovani-induced IL-10 production by DCs and that this DC-derived IL-10, in turn, suppresses the activation and maturation of DCs.

## DISCUSSION

DCs are key initiators of antileishmanial immune responses (11). However, it is not yet clear whether L. donovani activates or inhibits DCs. While some groups have reported that L. donovani infection promotes the activation and maturation of DCs, others have demonstrated an inhibitory effect of L. donovani on DC activation/maturation (14, 15, 17, 18). These conflicting reports compelled us to reassess the regulatory effects of L. donovani on DCs. Our present findings demonstrated that infection of BMDCs with LDPm or LDAm inhibited LPS-induced secretion of proinflammatory cytokines and upregulation of costimulatory molecule expression. However, L. donovani alone (without LPS treatment) had no effect on proinflammatory cytokine secretion or costimulatory molecule expression by BMDCs. Based on the latter finding, it should not be concluded that L. donovani enters DCs in an immunologically quiescent manner. Rather, our other data showed here that L. donovani exhibited the ability to induce IL-10 secretion from DCs (described below). We further demonstrated that sDCs from L. donovani-infected mice, compared to uninfected mice, displayed lower expression of costimulatory molecules and proinflammatory cytokines despite LPS stimulation. Although BMDCs and sDCs are not exact equivalents to one another, our above-mentioned findings at least ruled out the possibility that the L. donovani-induced inhibition was intrinsic to BMDCs. Thus, our results confirmed the inhibitory effect of L. donovani on DCs. Notably, the molecular mechanism by which L. donovani immunoregulates DCs is poorly understood. Our work has revealed that TIM-3 acts as a key receptor for mediating L. donovani-induced inhibition of DCs. Furthermore, we have identified the molecular pathway through which TIM-3 promotes DC inhibition in response to L. donovani infection.

Our data that TIM-3 is essential for DC inhibition induced by L. donovani document a new role for the TIM-3 receptor in DC immunoregulation during *Leishmania* infection. When TIM-3 expression was suppressed, DCs became resistant to L. donovani-induced inhibition *in vitro*. Similarly, *in vivo* blockade of TIM-3 with anti-TIM-3 antibody substantially increased the capacity of sDCs of L. donovani-infected mice to express costimulatory molecules and proinflammatory cytokines in response to LPS stimulation. The latter finding demonstrates the *in vivo* requirement of TIM-3 for DC suppression occurring during L. donovani infection. Although in this regard we are not ignoring the possibility that TIM-3 might have indirectly promoted L. donovani-induced DC suppression by regulating the function of other cells associated with DC immunoregulation, it is very much likely that TIM-3 expressed on the DC surface also played a role in directly mediating the inhibitory effects of L. donovani on DCs. In fact, our above-described *in vitro* results using BMDCs support the notion that DC-expressed TIM-3 directly mediates DC inhibition in response to L. donovani infection. This is in agreement with our previous report (23) demonstrating a general inhibitory role for TIM-3 in DC activation and maturation. Our results have also provided evidence that TIM-3-mediated L. donovani-induced inhibition of DCs serves a major role in suppressing antileishmanial T cell responses. For instance, silencing TIM-3 expression in DCs and thereby blocking the inhibitory effect of L. donovani on DCs considerably increased the antileishmanial type 1 T cell responses *in vivo*. These data indicate the therapeutic potential of targeting TIM-3 to augment antileishmanial immunity. In fact, we have shown here that treatment with anti-TIM-3 antibody reduces parasite burden in L. donovani-infected mice. Thus, our results document a previously unidentified role for TIM-3 in mediating L. donovani-induced inhibition of DCs and antileishmanial immune responses.

Analyses of the molecular mechanism by which L. donovani suppressed DC activation and maturation via TIM-3 revealed that L. donovani in fact triggered TIM-3 phosphorylation in DCs. The L. donovani-induced TIM-3 phosphorylation, however, was prevented by blocking TIM-3 with anti-TIM-3 antibody. Similarly, L. donovani separated from DCs by a cell-impermeable membrane failed to trigger TIM-3 phosphorylation in DCs. These results provide the first evidence that L. donovani activates TIM-3 in DCs possibly via a direct interaction. At present, we are uncertain about the identity of the TIM-3-interacting molecule (or molecules) present on the L. donovani surface, and we are currently investigating this aspect. In addition, efforts are ongoing to determine whether any other *Leishmania* species or pathogen can trigger TIM-3 phosphorylation in DCs. Nevertheless, our findings demonstrated the TIM-3-inducing ability of L. donovani in DCs. Our data also showed that L. donovani inhibited NF-κB signaling in DCs and that TIM-3 was required for this process. This finding correlated with our aforementioned observations that L. donovani suppressed DC activation/maturation in a TIM-3-dependent manner. Previous studies have shown that blockade of NF-κB activation alone can stall the activation/maturation of DCs (33–35). Based on these reports coupled with our results, we conclude that L. donovani suppresses DC activation and maturation by blocking NF-κB signaling via TIM-3. Interestingly, we observed that both LDPm and LDAm markedly inhibited LPS-induced DC activation/maturation at 24 h postinfection despite the fact that LDPm requires time for transformation into the LDAm form. A possible explanation for this is that LDPm can trigger TIM-3 phosphorylation even by interacting with DCs extracellularly (i.e., when amastigotes are not formed). The latter conclusion can be supported by our observation that TIM-3 phosphorylation was augmented in DCs as early as 2.5 min after incubation with LDPm, the time point at which parasite internalization does not occur (46). Accordingly, the lag time due to promastigote transformation into amastigote form may not influence DC inhibition kinetics. Anyway, our data collectively underscore the importance of TIM-3 in L. donovani-induced inhibition of DCs.

While exploring the TIM-3 signaling events induced by L. donovani, we observed that Btk interacted with TIM-3 and became activated within 2.5 min after incubation with L. donovani parasites. In addition, L. donovani-induced Btk activation was found to be TIM-3 dependent. These findings suggest that Btk participates in L. donovani-driven TIM-3 signaling in DCs. Our results further showed that Btk was necessary for L. donovani-induced inhibition of the NF-κB signaling and DC activation and maturation mediated via TIM-3. When Btk was silenced, L. donovani could not inhibit NF-κB signaling and subsequent DC activation and maturation. Thus, acting as an important component of L. donovani-induced TIM-3 signaling events, Btk imparts the inhibitory effect of L. donovani on DCs and thereby contributes to the suppression of antileishmanial immunity. In fact, a recent study using a murine model of experimental VL has demonstrated that ibrutinib (an inhibitor of both Btk and IL-2-inducible kinase [Itk]) efficiently cures L. donovani infection by enhancing the protective immune response (47). Although the latter study aligns with our current findings, this report by the Satoskar group did not clarify whether ibrutinib augmented antileishmanial immune responses by targeting specifically Btk or Itk. Furthermore, the role of Btk in the immunobiology of *Leishmania* infections is not yet well addressed. To date, only a few studies using X-linked immunodeficient (Xid) mice (which carry a mutation in the Btk gene) have demonstrated that Btk, depending on parasite species, contributes to either resistance or susceptibility to *Leishmania* infection. For instance, Xid mice have been shown susceptible to Leishmania amazonensis infection but resistant to Leishmania chagasi or Leishmania major infection (48–50). However, it is currently unknown whether and how *Leishmania* regulates Btk activity. Furthermore, the molecular mechanism by which Btk mediates the immunoregulatory effect during *Leishmania* infection has remained undefined. Here, our findings document the ability of L. donovani to induce Btk activation in DCs via TIM-3 and also demonstrate a previously unidentified role for Btk in L. donovani-mediated DC suppression by impairing NF-κB signaling.

Our results further suggest that L. donovani-induced TIM-3–Btk signaling suppresses NF-κB-driven activation and maturation of DCs by promoting IL-10 secretion. IL-10 is an immunosuppressive cytokine that is generally expressed at elevated levels in serum and lesional tissue of advanced VL patients (51). The role of IL-10 in the pathogenesis of VL is now well established. For instance, IL-10 is known to promote intracellular replication and persistence of L. donovani parasites, which favor disease progression (51). Neutralization of IL-10 with anti-IL-10 antibody, on the other hand, facilitates L. donovani clearance (52). Likewise, *IL-10*-deficient mice are found highly resistant to L. donovani infection (53). Together, these reports suggest a disease-promoting role for IL-10 in VL. Although some cellular sources (such as T cells and monocytes/macrophages) of IL-10 have been identified in VL (8, 38, 54), our previous (9) and current findings demonstrate that DCs also produce IL-10 in response to L. donovani infection. In fact, a recent report has also shown that DCs serve both as an important cellular source for IL-10 and as a major IL-10 responder during experimental VL, and thus influence the disease outcome significantly (45). However, the latter report did not address whether IL-10 produced during L. donovani infection exerts any effect on DCs. In addition, the receptor (or receptors) and the signaling pathway mediating L. donovani-induced IL-10 production by DCs are not fully understood. As mentioned above, our *in vitro* data have shown that IL-10 produced by DCs following L. donovani infection suppresses DC activation and maturation by blocking NF-κB signaling. Importantly, in the *in vivo* context, similar effects may also be mediated by IL-10 produced by other cells (e.g., T cells [38]) in response to L. donovani infection. Yet, our findings have demonstrated a key role for DC-derived IL-10 in L. donovani-induced inhibition of DCs. Our results have also identified the TIM-3 receptor and its downstream effector Btk as critical mediators of L. donovani-induced IL-10 secretion from DCs. Currently, the role of TIM-3 and Btk in regulating IL-10 expression has remained controversial. While some reports have suggested an inhibitory role for TIM-3 in IL-10 production in T cells and monocytes/macrophages (55, 56), others have proposed TIM-3 as an inducer of IL-10 expression in Treg cells and macrophages (57, 58). Similarly, Btk has been demonstrated as both a positive and a negative regulator of IL-10 expression in DCs (59, 60). In this regard, our findings have depicted TIM-3 and Btk as positive regulators of L. donovani-stimulated IL-10 production by DCs. It is possible that both TIM-3 and Btk play distinct role in the regulation of IL-10 expression depending on the kind of stimulation and the cell types.

Finally, an intriguing question that could be raised is how to relate the rapid kinetics of TIM-3 phosphorylation with the late DC inhibition observed *in vitro* during L. donovani infection. This can be explained by our data suggesting that the inhibition of DC activation and maturation by L. donovani depends on IL-10 production mediated by TIM-3 in DCs. Although we observed a rapid TIM-3 phosphorylation upon incubating DCs with L. donovani parasites, TIM-3 possibly triggered a cascade of signaling events that eventually led to IL-10 production by DCs (which started at 6 h postinfection and reached a peak level at 24 to 36 h) and thereby inhibited DCs. One of the key proximal mediators through which L. donovani-induced TIM-3 promoted IL-10 production in DCs was found to be Btk. However, similar to TIM-3 phosphorylation, Btk activation was also upregulated at 2.5 to 5 min upon incubation with LDPm. Therefore, it is very much likely that many other signaling molecules (at downstream of Btk) are also involved in mediating IL-10 production. Identification of these signaling molecules is currently underway. Nevertheless, IL-10 produced in this way inhibited the LPS-induced NF-κB signaling pathway and subsequent activation and maturation of DCs. Interestingly, we observed that maximum L. donovani-induced DC inhibition occurred at 24 h postinfection, the time point at which IL-10 was also maximally produced by DCs. Thus, even though TIM-3 is rapidly activated by L. donovani, the inhibition of DCs occurs late because it is dependent on TIM-3-mediated induction of IL-10 production by DCs possibly via a prolonged signaling cascade.

**FIG 10:**
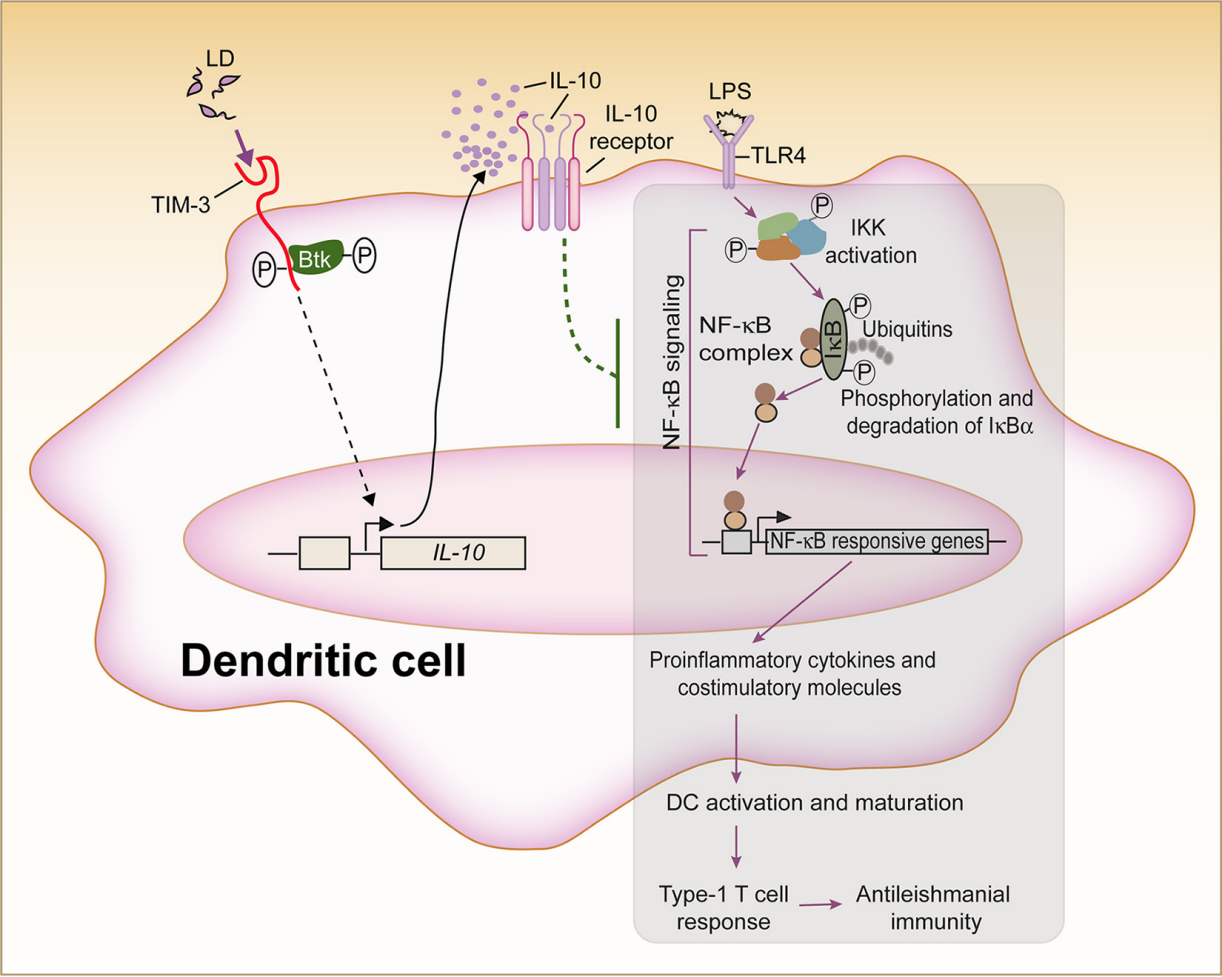
Model describing how TIM-3 controls antileishmanial immunity by immunoregulating DCs. Our results have identified a TIM-3-dependent mechanism for L. donovani-induced DC suppression. Briefly, we have shown here that L. donovani triggers TIM-3–Btk signaling, which promotes IL-10 secretion from DCs. IL-10 then inhibits the activation and maturation of DCs by blocking the NF-κB signaling pathway. The TIM-3-mediated L. donovani-induced inhibition of DCs subsequently impedes host-protective type 1 T cell responses. Thus, acting as a critical mediator of DC inhibition by L. donovani, TIM-3 plays a crucial role in regulating antileishmanial immunity. The gray-shaded area shows the events (which include NF-κB-driven DC activation and maturation, type 1 T cell response, and antileishmanial immunity) blocked by L. donovani-induced TIM-3 signaling.

In summary, the current study has identified a pivotal role for TIM-3 in inhibition of DCs and antileishmanial immunity during L. donovani infection. TIM-3 mediates the inhibitory effect of L. donovani on DCs via the downstream effector Btk, which blocks NF-κB signaling and the subsequent activation and maturation of DCs by promoting IL-10 secretion. This TIM-3-mediated L. donovani-induced inhibition of DCs eventually impedes type 1 T cell responses and thus contributes to the suppression of antileishmanial immunity (Fig. 10). Overall, our work documents a unique immunosuppressive mechanism by which L. donovani inhibits host immune responses. In light of our above findings, it seems likely that blocking the inhibitory effects of TIM-3 and thereby augmenting the immunostimulatory capacity of DCs can be a promising strategy for treating VL. Moreover, the present study will help to advance the understanding of TIM-3-mediated regulation of innate immune responses against other infectious diseases as well.

## MATERIALS AND METHODS

### Ethics statement.

Usage of L. donovani for the present study was approved by the Biosafety Committee of the Institute of Microbial Technology (IMTECH/IBSC/2015/16, CSIR-IMTECH/IBSC/2018/23, and CSIR-IMTECH/IBSC/2019/09). All animal experiments were done following the National Regulatory Guidelines issued by CPCSEA (Committee for the Purpose of Control and Supervision of Experiments on Animals), Government of India, and with approval of the Institutional Animal Ethics Committee of the Institute of Microbial Technology (IAEC/17/04 and IAEC/19/18).

### Reagents.

For immunoblot analysis, the following antibodies were used: anti-IκBα (sc-371), anti-β-actin (sc-47778), anti-IKKα (sc-7182), anti-IKKβ (sc-34673), and horseradish peroxidase (HRP)-labeled anti-goat IgG (sc-2354) (all from Santa Cruz Biotechnology); anti-phospho-IKK (Ser180/181, SAB4301429; Sigma-Aldrich); anti-phospho-Tyr (9411S), anti-TIM-3 (83882S), anti-Btk (8547S), and anti-phospho-Btk (Tyr223, 5082S) (all from Cell Signaling Technology); and HRP-labeled anti-rabbit IgG (HAF008) and anti-mouse IgG (HAF018) (both from R&D Systems). For immunoprecipitation assays, the reagents and antibodies used are listed below: protein A/G Plus-agarose beads (sc-2003; Santa Cruz Biotechnology), anti-TIM-3 (14-5871-85, clone 8B.2C12; eBioscience), and rat IgG1,κ (400402; BioLegend). The following antibodies were used for flow cytometry: phycoerythrin (PE)-labeled anti-CD40 (124610), fluorescein isothiocyanate (FITC)-labeled anti-CD80 (104706), FITC-labeled anti-CD86 (105006), FITC-labeled anti-CD11c (117306), allophycocyanin (APC)-labeled anti-CD11c (117310), APC-labeled anti-CD3 (100236), FITC-labeled anti-CD4 (100510), peridinin chlorophyll protein (PerCP)/Cyanin5.5-labeled anti-CD4 (100434), FITC-labeled anti-CD8 (100706), PE-labeled anti-TNF-α (506305), and PE-labeled anti-IL-10 (505008) (all from BioLegend); PerCP/Cyanin5.5-labeled anti-CD8 (551162) and APC-labeled anti-IL-12 (554480) (both from BD Biosciences); and PE-labeled anti-IFN-γ (12-7311-83) and PerCP/Cyanin5.5-labeled anti-F4/80 (45-4801-82) (both from eBioscience). Neutralizing anti-IL-10 (505012; clone JES5-16E3) and rat IgG2b,κ (400643; isotype control antibody) were purchased from BioLegend. Recombinant mouse granulocyte-macrophage colony-stimulating factor (GM-CSF) and IL-4 were purchased from Peprotech. The On-TargetPlus nontargeting control pool siRNAs and SMARTpool siRNAs targeting TIM-3 or Btk were obtained from Dharmacon. Notably, these siRNAs were used in previous studies as well (23, 24, 61). The *in vivo* anti-TIM-3 monoclonal antibody (BE0115, clone RMT3-23) and corresponding isotype control rat IgG2a,κ (BE0089) were purchased from BioXcell. LPS (Escherichia coli O111:B4), polystyrene latex beads (2.0-μm mean particle size; L3030), and other reagents were obtained from Sigma-Aldrich.

### Animals and L. donovani parasites.

BALB/c mice and golden hamsters (Mesocricetus auratus) were bred and maintained under pathogen-free conditions at the animal house facility of the Institute of Microbial Technology. The L. donovani strain AG83 (MHOM/IN/83/AG83; American Type Culture Collection [ATCC PRA-413]) was maintained in golden hamsters (62). Amastigotes were derived from spleens of infected hamsters as mentioned previously (63). After that, amastigotes were transformed into promastigotes and cultured as described previously (64).

### Preparation of SLA and heat-killed L. donovani parasites.

SLA were prepared from LDPm (10^9^/mL) as described previously (9, 65). Heat-killed L. donovani parasites were obtained by incubating LDPm at 56°C for 10 min (66).

### DC generation, infection with L. donovani parasites, and treatments.

BMDCs were generated from bone marrow precursors of male or female BALB/c mice (8 to 12 weeks old) as described previously (67). DCs (5 × 10^6^/well) were subsequently infected with LDPm (stationary phase) or LDAm *in vitro* at a parasite-to-DC ratio of 10:1 for specified times in RPMI 1640 complete medium (10% fetal bovine serum [FBS], l-glutamine, nonessential amino acids, sodium pyruvate, penicillin-streptomycin, and 2-mercaptoethanol). Approximately 78% ± 3.5%, 84% ± 2%, and 86% ± 2.6% of DCs were found infected when incubated with LDPm for 6 h, 12 h, and 24 h, respectively (see Fig. S5B in the supplemental material). DCs were then washed and treated with LPS (500 ng/mL) for the indicated times.

In some experiments, DCs were treated with anti-TIM-3 antibody prior to LDPm infection. Briefly, DCs (5 × 10^6^/well) were incubated with anti-mouse FcγIII/II (BD Bioscience) for 0.5 h at 37°C to block Fc receptor binding. DCs were then treated for 1 h at 37°C with 10 μg of anti-TIM-3 antibody (clone RMT3-23) or rat IgG2a,κ (isotype-matched control immunoglobulin). In some other experiments, DCs (5 × 10^6^/well) were infected with LDPm for 24 h in the presence or absence of 10 μg/mL neutralizing anti-IL-10 antibody or rat IgG2b,κ (isotype-matched control immunoglobulin).

### EMSA and immunoblot analysis.

Nuclear and cytoplasmic extracts of DCs were made as mentioned previously (68). EMSA was done using a ^32^P-labeled DNA probe containing NF-κB-binding sites obtained from major histocompatibility complex class I (MHC-I) H2K promoter, 5′-CAGGGCTGGGGATTCCCCATCTCCACAGTTTCACTTC-3′, or a double-stranded OCT-1 DNA probe (control), 5′-TGTCGAATGCAAATCACTAGAA-3′ (23). Bands were visualized using a phosphorimager (PharosFX molecular imager; Bio-Rad). Immunoblot analysis was carried out as illustrated previously (44). Densitometry quantification was done using Scion Image software (Scion Corporation).

### Assessment of L. donovani-induced TIM-3 phosphorylation.

BMDCs were plated at 5 × 10^6^ cells/well with 1 mL of RPMI 1640 complete medium in a 24-well low-cluster plate and incubated with live LDPm at a parasite-to-DC ratio of 10:1 for specified times (i.e., 2.5 to 60 min). In some experiments, BMDCs were incubated for 2.5 min with heat-killed LDPm (at a similar parasite-to-DC ratio) or polystyrene latex beads (10 μL). Alternatively, BMDCs were incubated for 2.5 min with LDPm separated by a 0.4-μm transwell insert (Corning [69]; BMDCs were kept in the lower wells and LDPm in the upper well]. DCs were then chilled on ice, resuspended in 1 mL of protein cross-linker solution (dimethyl 3,3-dithiopropionimidate dihydrochloride [Sigma-Aldrich] in PBS [2 mg/mL]), and kept at room temperature for 20 min (34). Afterward, DCs were lysed as mentioned previously (23). Approximately 800 μg of whole-DC lysates was subjected to immunoprecipitation using anti-TIM-3 antibody (clone 8B.2C12; eBioscience) or control immunoglobulin (rat IgG1,κ [clone RTK2071; BioLegend]) as described previously (23). Immunoprecipitated proteins were then analyzed by immunoblotting.

To determine the attachment of LDPm on the BMDC surface during 2.5-min and 5-min incubation periods, LDPm and BMDCs were first labeled with carboxyfluorescein succinimidyl ester (CFSE; 5 μM; Sigma-Aldrich) and eFluor 670 (2.5 μM; eBioscience) dyes, respectively. BMDCs (5 × 10^6^/well) were then incubated with LDPm for these times as mentioned above and fixed with 4% paraformaldehyde, and the proportion of BMDCs with attached LDPm was determined by measuring the frequency of the CFSE^+^ eFluor 670^+^ population via flow cytometry. Next, to determine the number of parasites attached per DC during 2.5-min and 5-min incubation times, BMDCs (5 × 10^5^) were made to adhere to coverslips (18 mm) for 6 h and incubated with LDPm (parasite-to-DC ratio of 10:1) for the above-mentioned time points (i.e., 2.5 min and 5 min). Cells were then fixed with 4% paraformaldehyde and gently washed with PBS two times. Afterward, the number of LDPm adhering to individual BMDCs was determined by Giemsa staining.

### RNA-mediated interference.

Transfection of DCs with siRNAs (60 nM) was carried out using Lipofectamine RNAiMAX reagent (Invitrogen) as described previously (10, 23).

### Measurement of cytokines.

DCs (1 × 10^6^/mL) were infected with LDPm or LDAm for various times or left uninfected. DCs were then washed and stimulated with LPS for an additional 24 h. Alternatively, DCs were infected with LDPm or LDAm and then kept untreated (i.e., without LPS treatment). In some experiments, DCs were transfected with control siRNA or siRNA specific for TIM-3 or Btk prior to L. donovani infection or else infected with LDPm in the presence or absence of 10 μg/mL anti-IL-10 neutralizing antibody or isotype control antibody. Supernatants were assayed for IL-12p70 (555256; BD Biosciences), TNF-α (88-7324-88; Invitrogen), or IL-10 (88-7105-88; Invitrogen) using ELISA kits following the manufacturer’s instructions.

### Assessment of activation and maturation of sDCs obtained from L. donovani-infected mice.

BALB/c mice (4 to 6 weeks old) were injected intravenously with LDPm (2 × 10^7^/mouse) or left uninfected. On day 45 after LDPm infection, splenocytes derived from these L. donovani-infected mice or uninfected mice were treated (for 24 h) with LPS. The level of costimulatory molecules on sDCs was analyzed via flow cytometry after gating CD11c^+^ F4/80^−^ cells. To analyze intracellular expression of IL-12 and TNF-α, splenocytes were treated with LPS for 24 h. At the 20th hour of LPS stimulation, brefeldin A (10 μg/mL) was added. Splenocytes were then surface stained with anti-CD11c-FITC and anti-F4/80-PerCP/Cyanin5.5, followed by intracellular immunostaining using a fixation/permeabilization buffer kit (eBioscience; 88-8823-88) and anti-TNF-α-PE, anti-IL-12-APC, or respective isotype-matched control antibodies. Alternatively, to examine intracellular IL-10 expression, splenocytes were surface stained with anti-CD11c-APC and anti-F4/80-PerCP/Cyanin5.5, followed by intracellular staining using the fixation/permeabilization buffer kit and anti-IL-10-PE or isotype control antibody. The percentage of sDCs (CD11c^+^ F4/80^−^ cells) expressing IL-12, TNF-α, or IL-10 was determined by flow cytometry.

### *In vivo* anti-TIM-3 antibody treatment and analysis of activation/maturation of sDCs and parasite burden in L. donovani-infected mice.

BALB/c mice that had been infected with LDPm as described above were treated with anti-TIM-3 antibody (500 μg/mouse) or control immunoglobulin (Ctrl IgG; 500 μg/mouse) at 2 days prior to LDPm infection and subsequently (200 μg of control immunoglobulin or anti-TIM-3 antibody/mouse each time) on a weekly basis until 35 days postinfection. In this regard, the age-matched uninfected mice that were kept untreated served as experimental controls. After 45 days of L. donovani infection, the weight of spleens and livers of these mice and the parasite load in these organs were measured. The liver and splenic parasite load was assessed by the stamp-smear method and is expressed as Leishman-Donovan units (LDU) (65). Additionally, the splenocytes prepared from these mice were stimulated (or not) with LPS for 24 h. The expression of costimulatory molecules and cytokines such as IL-12, TNF-α, and IL-10 by sDCs was determined as described above.

### DC transfer experiments.

BALB/c BMDCs (1 × 10^6^) were infected for 24 h with LDPm at a parasite-to-DC ratio of 10:1. Under this condition, ~82% of DCs were infected with LDPm and approximately 5,240 intracellular amastigotes/1,000 DCs were observed (Fig. S5A). DCs were then treated with LPS for an additional 24 h. Treatment with LPS reduced the proportion of infected DCs to ~67% and the number of intracellular amastigotes/1,000 DCs to around 3,630 (Fig. S5A). Alternatively, DCs were only treated with PBS or LPS for 24 h. In some cases, DCs were transfected (or not) with control siRNA or TIM-3-specific siRNA prior to LDPm infection. DCs (1 × 10^6^) were subsequently injected intravenously into L. donovani-infected BALB/c mice (on the 15th, 25th, 35, and 45th days after L. donovani infection). On the 48th day postinfection, the spleens and livers were removed from these mice to measure their weights and the parasite load. In addition, splenocytes (1 × 10^6^/well) derived from these mice were subsequently stimulated with SLA (80 μg/mL) for 48 h or left unstimulated (40, 41), and the percentages of splenic CD4^+^ and CD8^+^ T cells expressing type 1 (IFN-γ and ΤΝFα) or type 2 (IL-10) cytokines were determined via flow cytometry. To detect type 1 and type 2 cytokine expression in CD4^+^ and CD8^+^ T cells, splenocytes were surface stained with anti-CD3-APC along with anti-CD4-FITC (or -PerCP/Cy5.5), anti-CD8-PerCP/Cy5.5 (or -FITC), or the respective isotype control antibody. Splenocytes were then subjected to intracellular staining using the fixation/permeabilization buffer kit (mentioned above) and PE-labeled anti-IFN-γ, anti-TNF-α, anti-IL-10, or the respective isotype control antibody. The percentage of IFN-γ-, TNF-α-, or IL-10-expressing CD4^+^ or CD8^+^ T lymphocytes within the gated CD3^+^ cell population was assessed by flow cytometry.

For another DC adoptive transfer experiment, BMDCs (1 × 10^6^) were infected with LDPm for 24 h as described above. DCs were then treated (or not) with LPS for 24 h and adoptively transferred into uninfected mice on days 0, 15, 25, and 35. On the 45th day, the spleens and livers were isolated, and the parasite burden in liver and spleen was assessed as illustrated above.

### Flow cytometry.

Flow cytometry was carried out with a C6 Accuri flow cytometer (BD Biosciences). The FlowJo software (Tree Star) was used for analysis of the data.

### Statistical analysis.

All statistical analyses were performed using one-way analysis of variance (ANOVA) (SigmaPlot 11.0 program). A *P* value of <0.05 was considered significant.
