# Supplementary figures and images for: Leishmania donovani Impedes Antileishmanial Immunity by Suppressing Dendritic Cells via the TIM-3 Receptor
Source: mBio. 2022 Aug 4;13(4):e03309-21. doi: 10.1128/mbio.03309-21 (PMC9426438; doi:10.1128/mbio.03309-21)

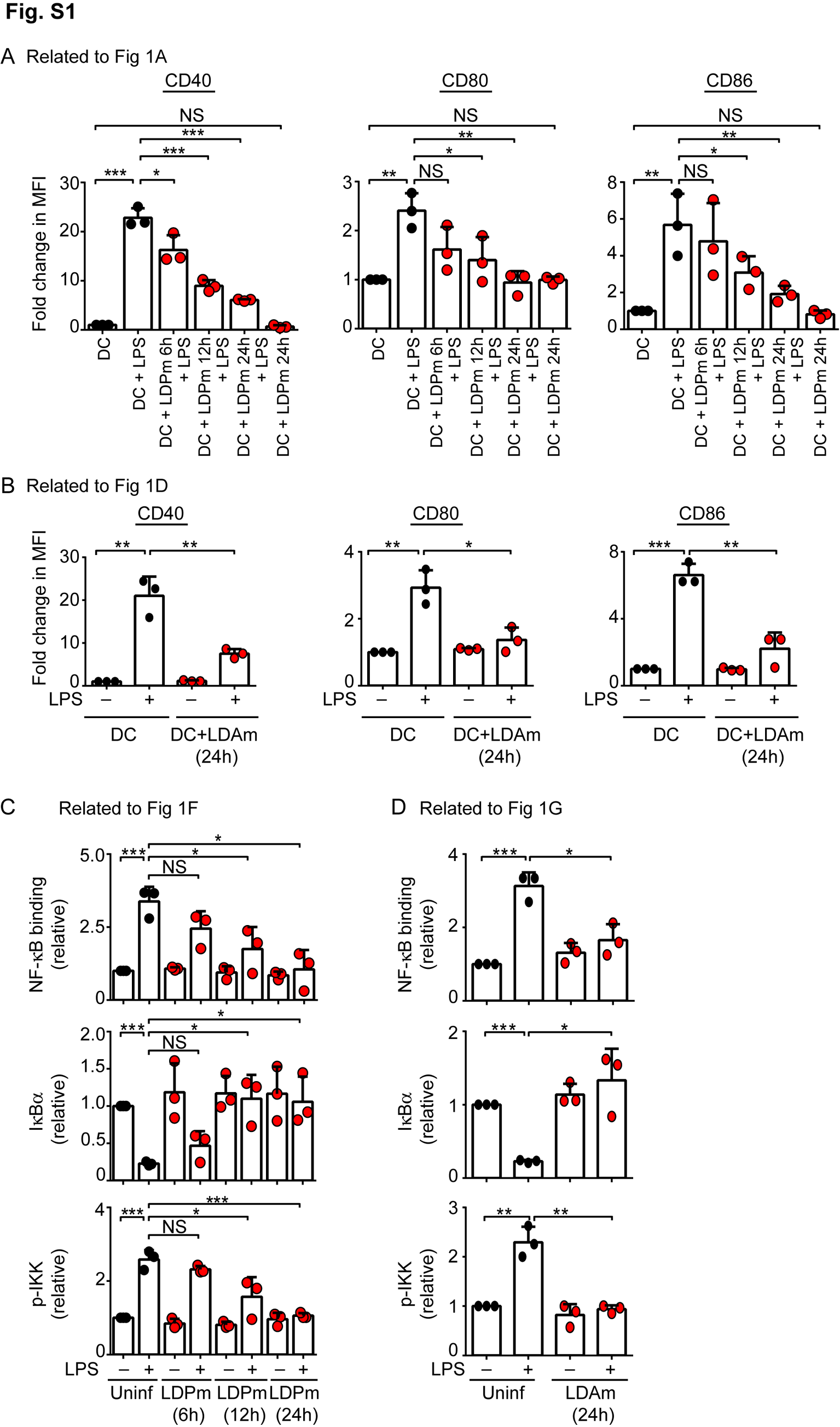

Supplement: FIG S1 [file mbio.03309-21-s0001.tif]

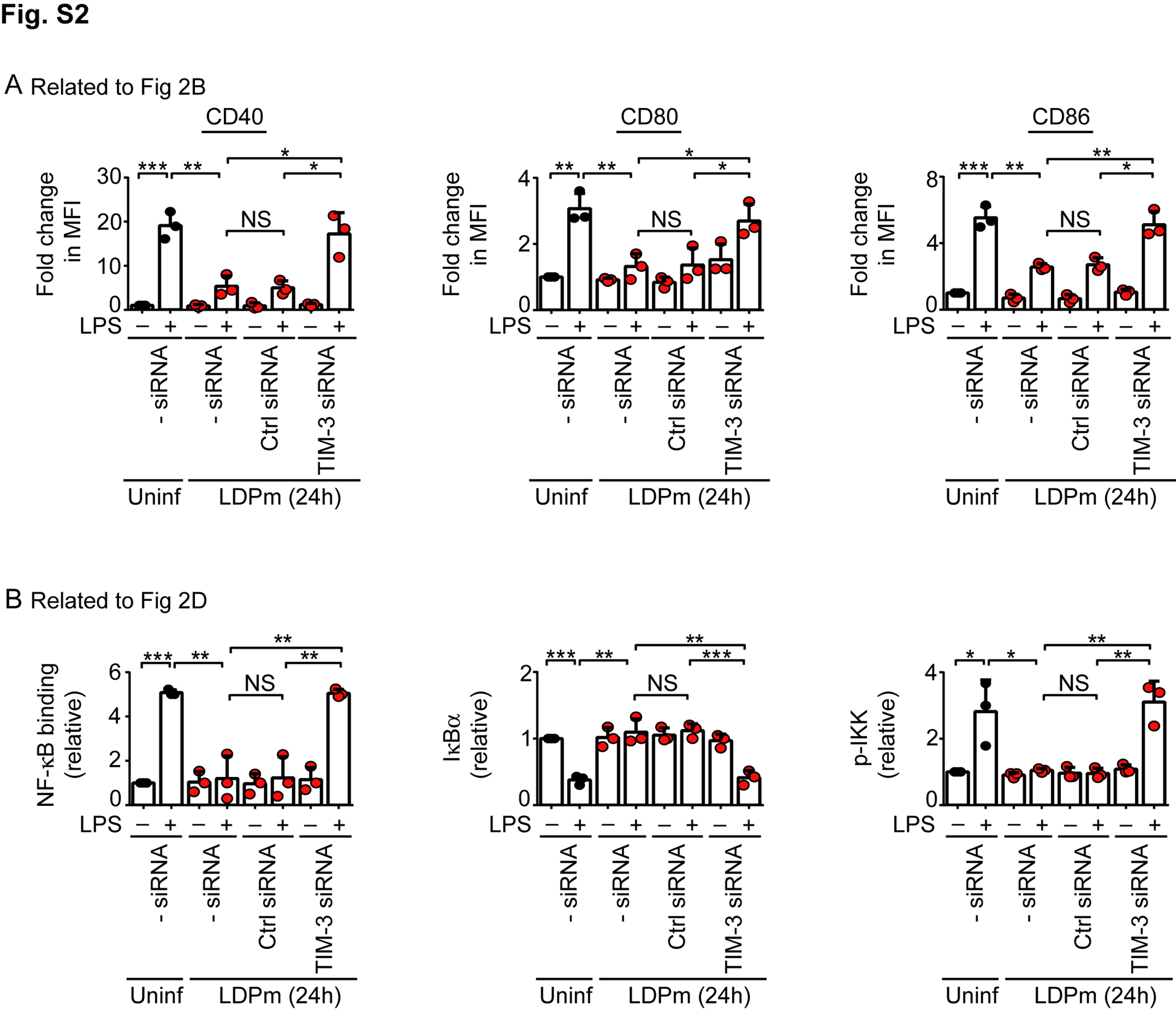

Supplement: FIG S2 [file mbio.03309-21-s0002.tif]

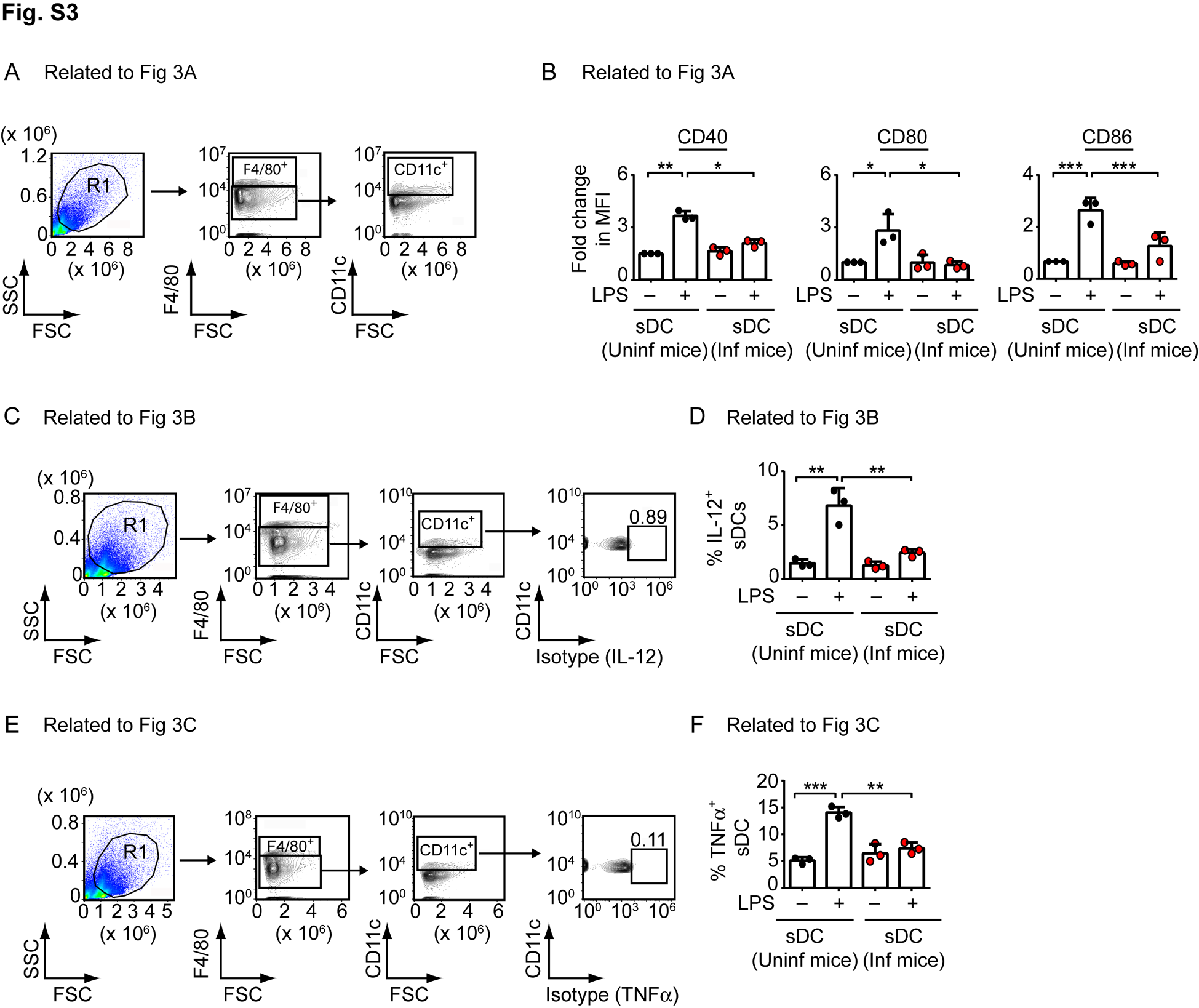

Supplement: FIG S3 [file mbio.03309-21-s0003.tif]

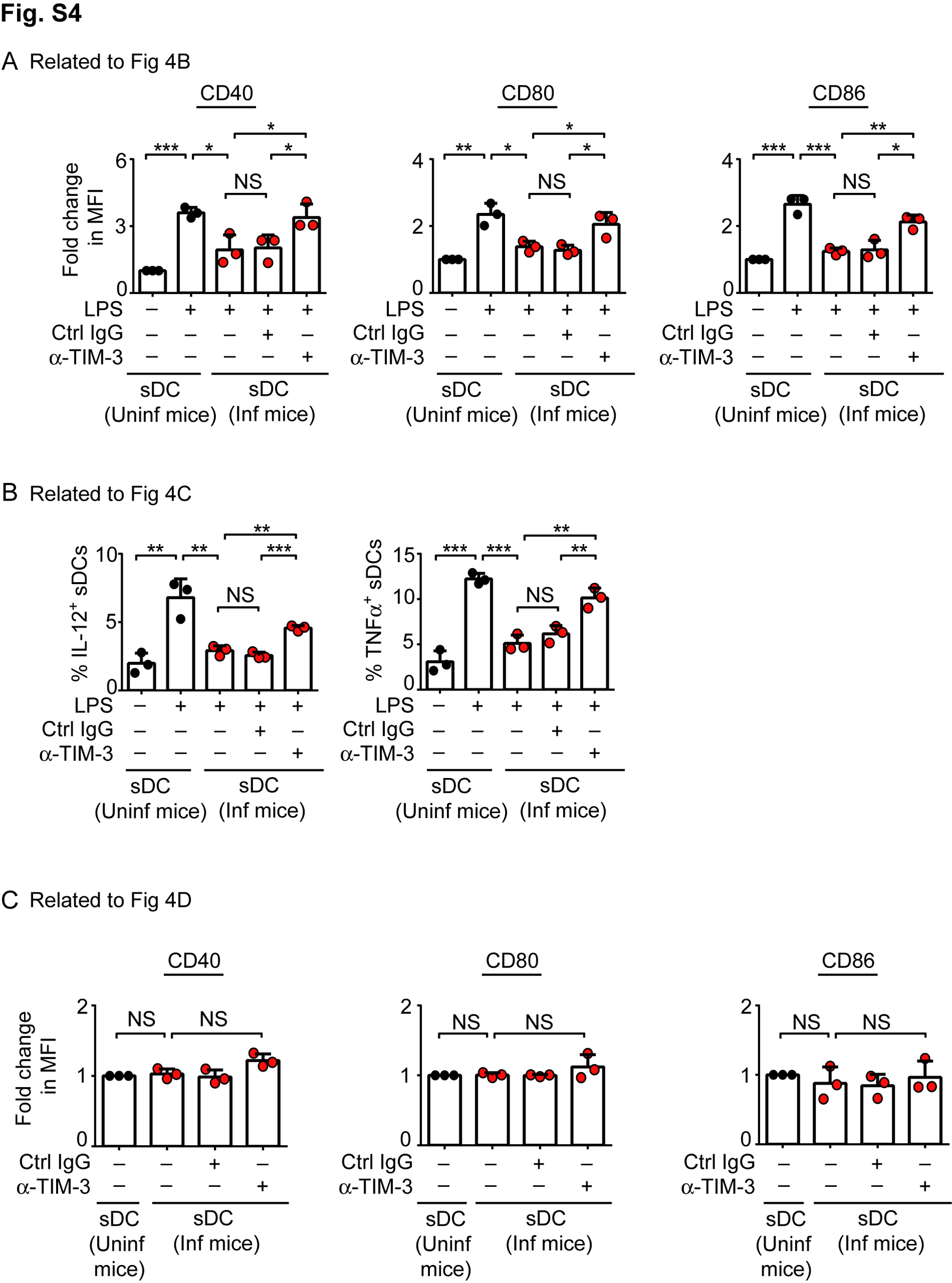

Supplement: FIG S4 [file mbio.03309-21-s0004.tif]

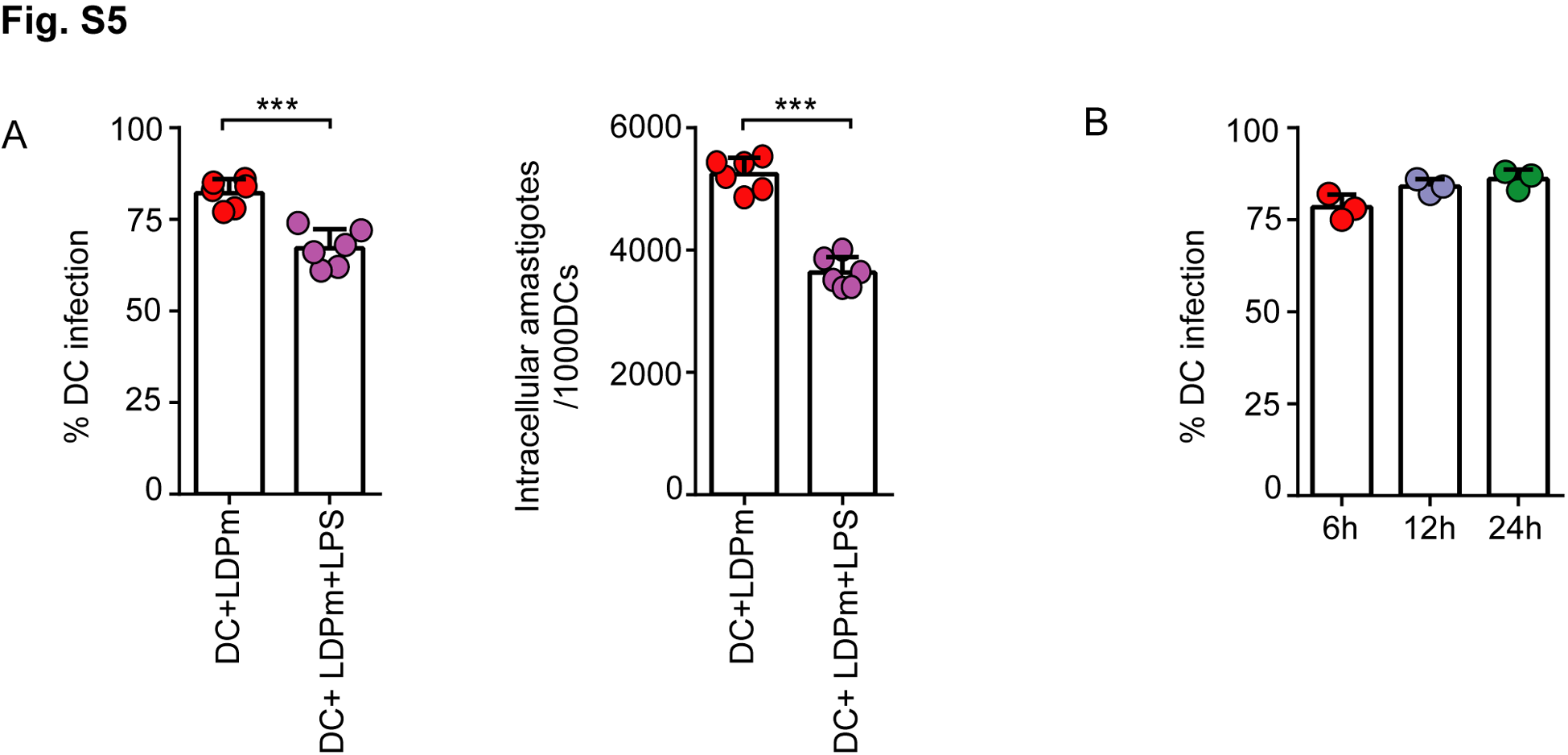

Supplement: FIG S5 [file mbio.03309-21-s0005.tif]

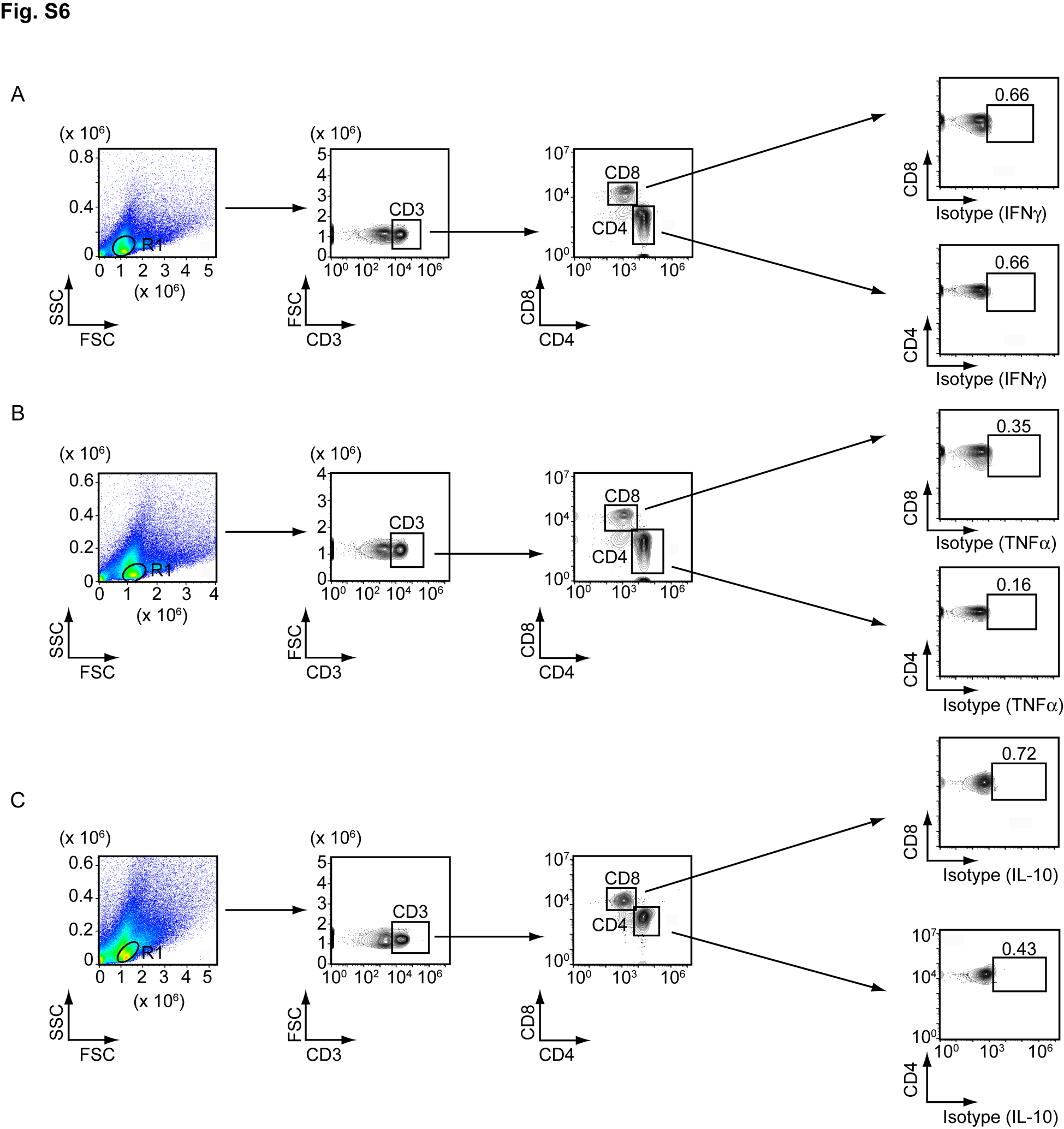

Supplement: FIG S6 [file mbio.03309-21-s0006.tif]

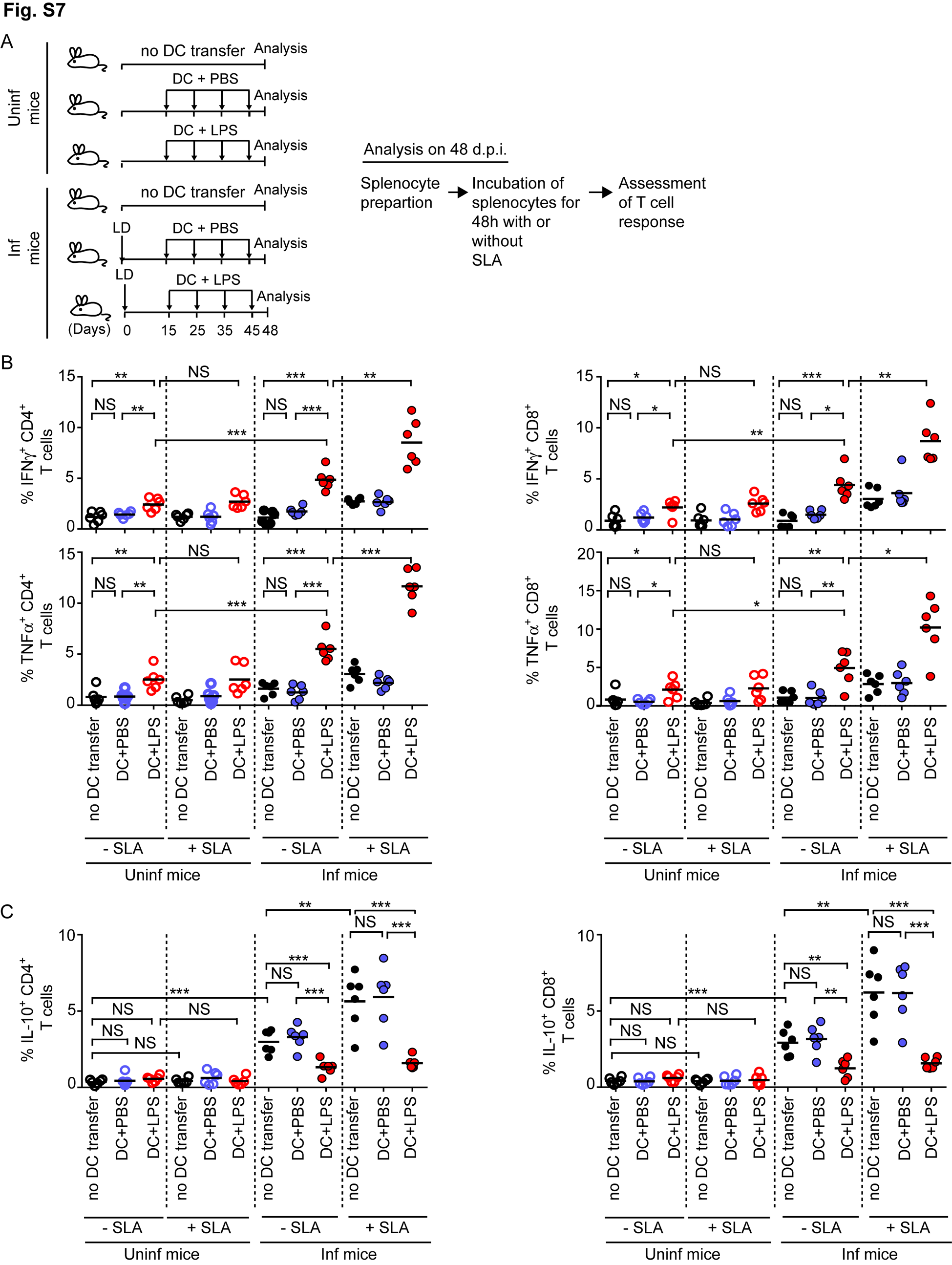

Supplement: FIG S7 [file mbio.03309-21-s0007.tif]

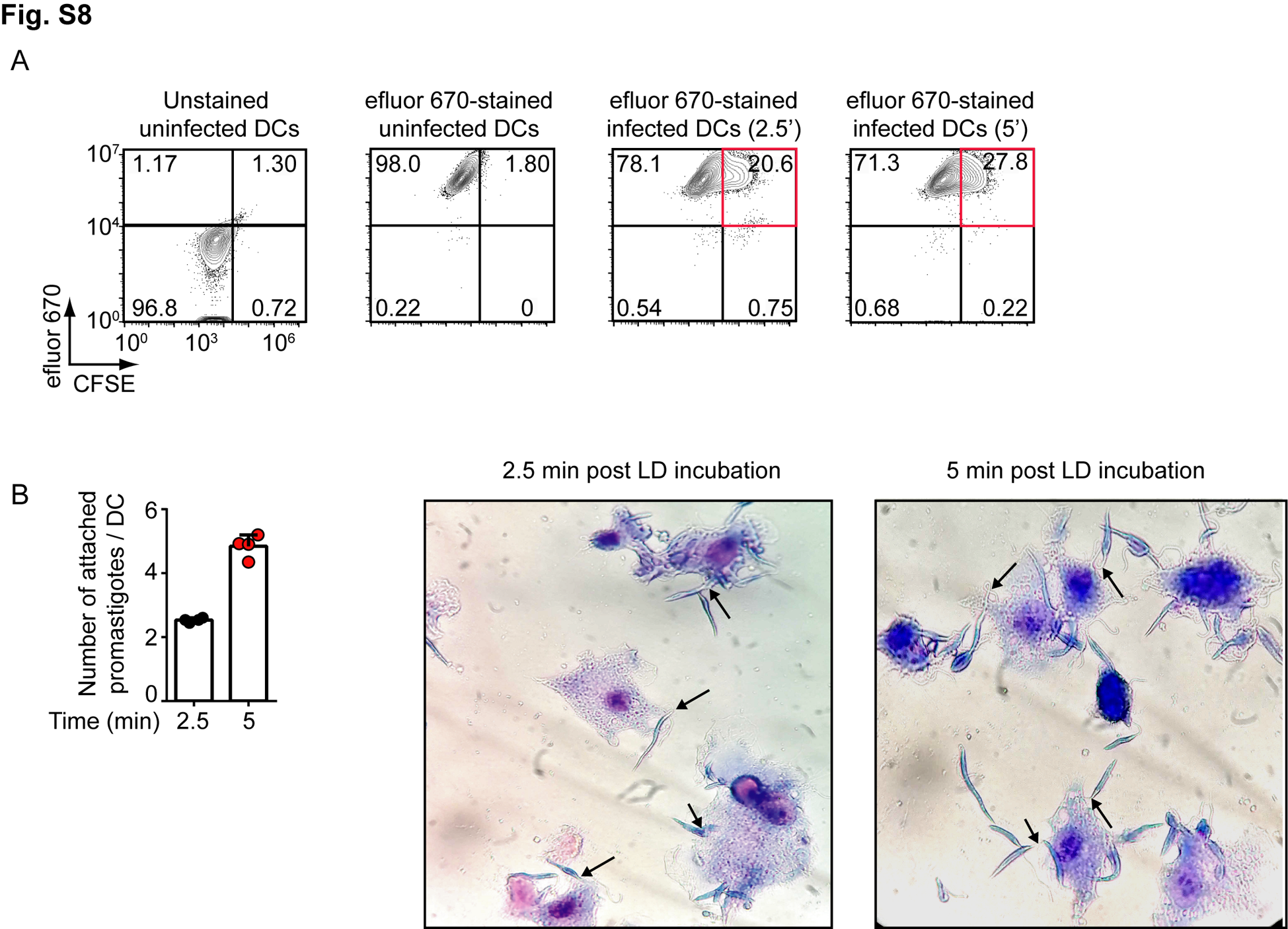

Supplement: FIG S8 [file mbio.03309-21-s0008.tif]

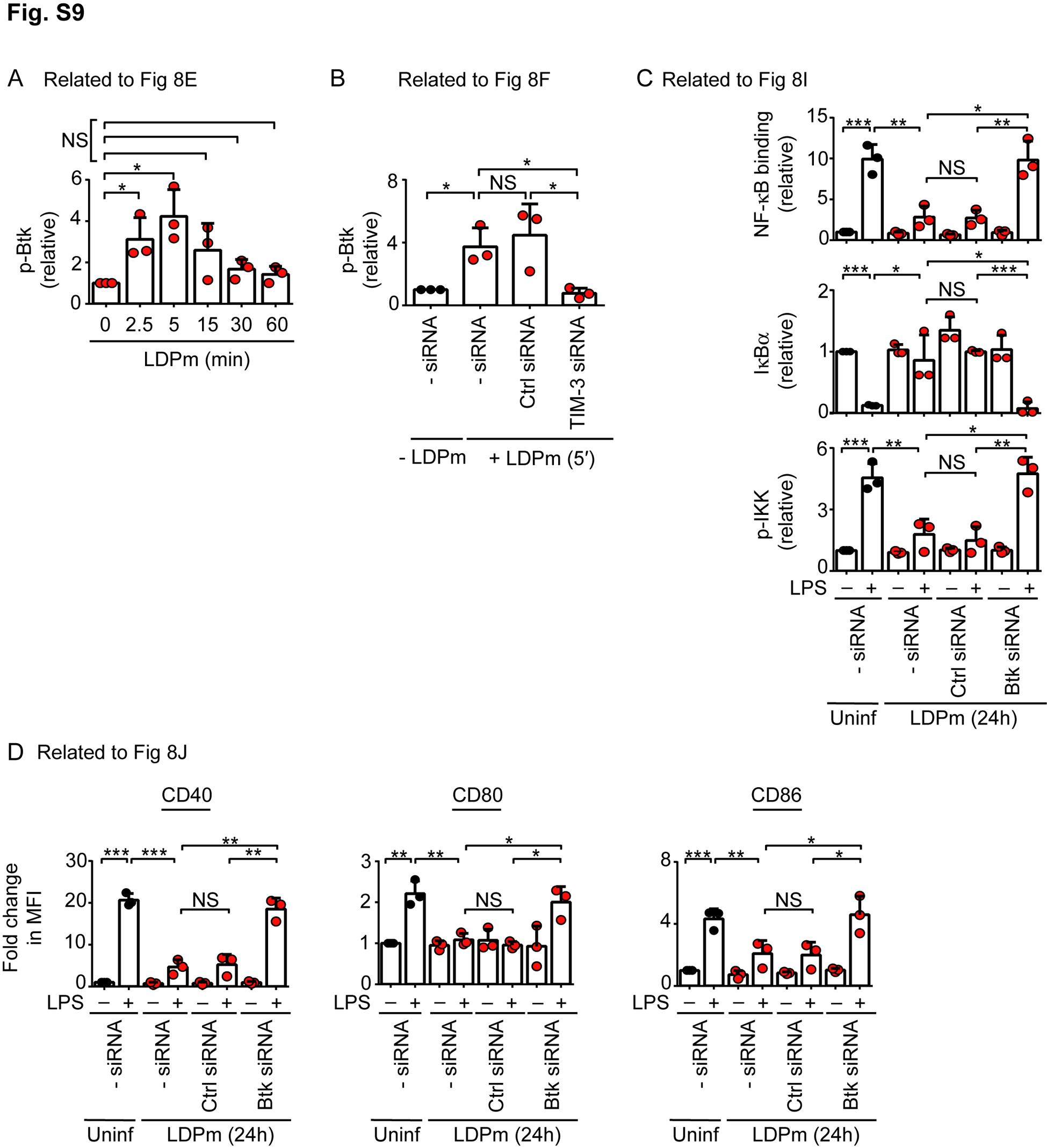

Supplement: FIG S9 [file mbio.03309-21-s0009.tif]

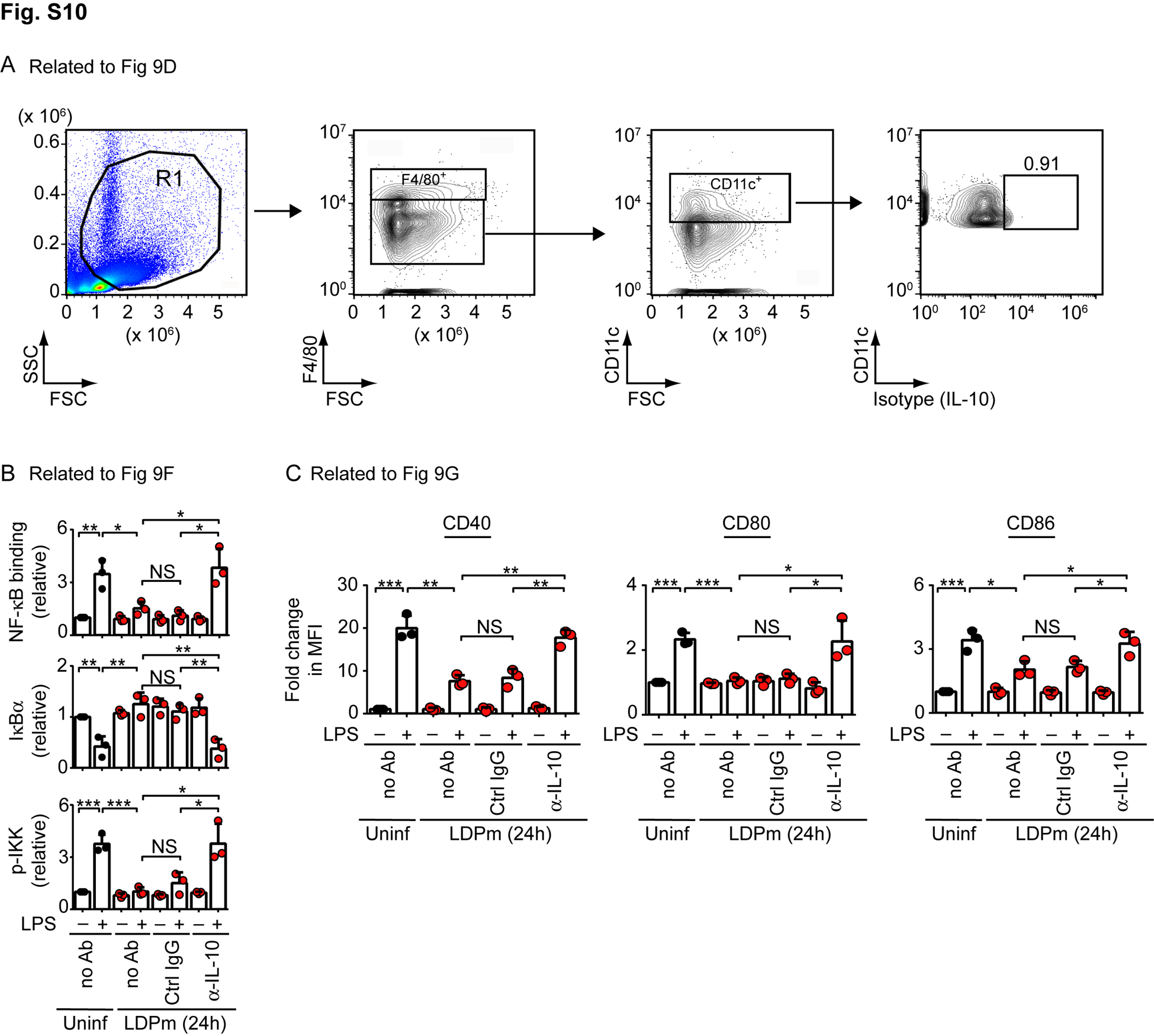

Supplement: FIG S10 [file mbio.03309-21-s0010.tif]
